# Ethnic disparities in opioid prescribing for cancer pain and associated emergency department visits and hospital admissions in the last three months of life: a retrospective cohort study

**DOI:** 10.1038/s41416-025-03200-4

**Published:** 2025-09-25

**Authors:** Emeka Chukwusa, Sophie Law-Clucas, Martin Gulliford, Sabrina Bajwah, Stephen Barclay, Rashmi Kumar, Gemma Clarke, Tanya Siriwimala, Jonathan Koffman

**Affiliations:** 1 Institute of Psychiatry, Psychology & Neuroscience, King’s College London, London, United Kingdom; 2 Cicely Saunders Institute, King’s College London, London, United Kingdom; 3 Wolfson Palliative Care Research Centre, Hull York Medical School, University of Hull, Hull, United Kingdom; 4 School of Population Health and Environmental Sciences, King’s College London, London, United Kingdom; 5 Department of Public Health and Primary Care, University of Cambridge, Cambridge, United Kingdom; 6 Academic Unit of Palliative Care, Leeds Institute of Health Sciences, University of Leeds, Leeds, United Kingdom; 7 Guy’s and St Thomas’ NHS Foundation Trust, London, United Kingdom

**Keywords:** Pain management, Palliative care

## Abstract

**Background:**

Ethnic inequalities in pain management at the end of life remain underexplored in the UK. We examined associations between patient ethnicity, opioid prescribing, and related healthcare use among cancer decedents.

**Methods:**

Retrospective cohort study including 232,329 adults (≥18 years) diagnosed with cancer between 2011 and 2021. Primary care records from the Clinical Practice Research Datalink Aurum were linked to hospital and mortality data. Person-time rates of opioid prescriptions, emergency department (ED) visits, and hospital admissions in the last three months of life were estimated. Poisson regression with Generalised Estimating Equations generated adjusted rate ratios (aRRs) and 95% confidence intervals (CIs), controlling for relevant covariates.

**Results:**

Of 3,987,635 opioid prescriptions, 620,232 (16%) occurred in the final three months. Prescription rates were highest among White patients (969.97–894.43/1000 person-months). Compared with White patients, prescribing was significantly lower among Black (aRR 0.91, 95% CI 0.87–0.95), South Asian (aRR0.93, CI0.89–0.97), Mixed (aRR 0.85, CI 0.79–0.92) and Other ethnic groups (aRR 0.90, CI 0.85–0.96). Patients from minority ethnic backgrounds, particularly Black and South Asian, more often experienced ≥2 ED visits and ≥2 hospital admissions.

**Conclusion:**

Minority ethnic patients with cancer receive fewer opioids and experience higher acute care use near the end of life. Tackling system-level inequities is critical to achieving pain management.

## Background

Pain affects up to two-thirds of patients with cancer, increasing to 80–90% of patients at the end of life [1–3]. Pain is highly distressing and debilitating for those who experience it. Many people who experience cancer pain state that it prevents them from performing previously taken-for-granted activities of daily living and enjoying life [4]. Opioids remain the first-choice analgesic for moderate to severe cancer pain and are highly successful in controlling pain for around 90% of patients with advanced cancer, many of whom are at the end of life [5, 6]. When used appropriately, opioids lead to clinically significant improvements in patients quality of life [7] and may even confer a survival benefit [8]. Opioids used to manage cancer represent a population quality indicator for end-of-life care ([9] and their use is endorsed by the *Lancet Commission on Pain Relief and Palliative Care*, which states that adequate pain relief is a human right [10]. However, inequities in prescribing opioids to manage cancer pain exist and have specifically been noted among patients from minority ethnic backgrounds. Evidence from recent systematic reviews [11, 12] identified and appraised single-site USA studies [13–16] where marked ethnic disparities were observed in the receipt of opioids to manage cancer pain, even after controlling for age, health insurance, gender, and pain intensity. Furthermore, unrelieved cancer pain is the most frequently recorded reason for using unscheduled GP out-of-hours care [17] and often results in emergency department (ED) visits [18, 19] and multiple unplanned hospital admissions [20].

England has become progressively more ethnically diverse [21] and recent evidence shows variation in cancer incidence across broad ethnic groups, with increasing incidence for certain cancers among some minority ethnic populations [22]. Whilst previous United Kingdom studies have evidenced ethnic disparities in end-of-life care for cancer patients [23] no studies have examined the association between patient ethnicity and the prescribing of opioids to manage cancer pain at the end of life, despite claims that this may be an issue in the NHS [24]. The COVID-19 pandemic exposed longstanding inequities in healthcare for people from minority ethnic backgrounds in the UK [25, 26]. If under-prescribing of opioids for cancer pain exists, it may represent another important, but previously overlooked health inequity. A better understanding of this issue is essential to optimise high-quality end of life care for all. Therefore, we examined for the first time the association between patient ethnicity and opioid prescribing in the last three months of life, when pain is known to increase [2]. We also evaluated this association with ED visits and hospital admissions.

## Methods

### Study design and data sources

We used primary care electronic health records from the Clinical Practice Research Datalink (CPRD) Aurum, a longitudinal anonymised database containing information on more than 14 million registered patients from primary care [27]. The CPRD Aurum comprises anonymised patient records from practices using the EMIS web software system [28]. Whilst it covers approximately 13% of the population of England, it is representative of the population in terms of geographical coverage and patient ethnicity [27, 28]. As of 2010, valid ethnicity is now being recorded for 90% of newly registered patients in primary care [29]. We conducted a population-based retrospective cohort study linking primary care records with Hospital Episode Statistics (HES) and Office for National Statistics (ONS) mortality records. In the UK, most patients are registered with a general practitioner (GP [family doctor]) and most opioids are prescribed in primary care [30]. Patients typically obtain all their repeat prescriptions (refill) from their GP. Hospital, oncology, hospice, and palliative care physicians provide medicines only for inpatients or for short periods (maximum two weeks) for outpatients or discharges. Prescriptions are recorded electronically and clinical data, including diagnoses, are documented using diagnostic codes. Only data that had undergone quality checks and met the CPRD criteria for data quality were used. This study is reported according to the Strengthening the Reporting of Observational Studies in Epidemiology (STROBE) guideline.

### Ethical approval

CPRD has overarching ethics approval from the Health Research Authority (HRA) to support research using anonymised patient data for public health benefit. Once CPRD receives anonymised data from a GP practice, the data is fully compliant with the Information Commissioner’s Office (ICO) anonymisation code of practice and patient privacy is protected. Requests by researchers to access the data are reviewed via the MHRA CPRD RDG process to ensure that the proposed research is of benefit to patients and public health Scientific approval for this study was granted by the CPRD Independent Scientific Advisory Committee (Ref: 21_000663) [31].

### Study Population

Our study population comprised adults (aged ≥18 years) (i) who died between 2011 and 2021; (ii) who had a diagnosis of malignant neoplasms; (iii) who were prescribed an opioid in the last three months of life, and (iv) who were eligible for linkage. We included all those with a diagnosis of lung, bowel, female breast, and prostate cancers (the UK’s four main causes of cancer death). We also included head and neck cancer, which is a high pain prevalence cancer [32], haematological cancers, specifically myeloma, which is more common in some ethnic groups and liver, stomach and cervical cancer, which have a higher incidence in some ethnic groups compared to the majority White ethnic group [22]. The diagnosis of cancer in CPRD was identified from diagnostic codes from previous studies [33]. The codes were independently verified by two clinicians working in palliative care (SB1) and general practice (SB2) who were part of the study team. In situations where more than one cancer type was recorded, the cancer recorded closest to the time of death was assumed as their primary cancer site, as this would more likely reflect the reason for prescribing opioids to manage pain or their use of health services (emergency department visits and hospital admissions) at the end-of-life. To reduce attrition bias, we included people who had been registered to a general practice located in England for at least one year and those who were registered with a general practice that contributed data to all study years.

### Study Variables

The main outcome of interest was the number of prescriptions of Step 2 opioids used to manage mild to moderate pain and Step 3 opioids used to manage moderate to severe pain, as recommended by the WHO Analgesic Ladder, in the final three months of life [34, 35]. All prescriptions of opioids were identified using codes from the British National Formulary (BNF). We included opioids (BNF: 04070200) and opioid-containing formulations. The selection of opioids was overseen by a palliative care pharmacist (See Appendix for opioids included).

Secondary outcomes included the number of multiple hospital admissions and the number of emergency department visits in the last three months of life. We defined “multiple hospital admissions” as having two or more admissions in the final three months of life for any reason. “Multiple emergency department visits” were defined as having two or more emergency department visits, excluding visits where the only hospital contact was an outpatient appointment or clinic [36].

Self-assigned patient ethnicity [37, 38] was the exposure variable, classified into five major groups and 16 disaggregated subcategories based on the previous study classifications that used the 2001 Census for England [39, 40]. The categories comprise: Black (Caribbean, African, Any other Black background), Mixed (White and Black Caribbean, White and Black Asian, White and Black African, Any Other Mixed background), Other (Chinese, Other), South Asian (Indian, Pakistani, Other) and White (British, Irish, any other White background). For individuals with more than one ethnicity recorded in their primary care records, we used a previously developed algorithm from the CPRD to assign a ‘single’ best ethnicity. This algorithm prioritises codes based on frequency and recency of recording [41]. In cases where multiple or conflicting ethnicity codes were recorded, the most frequently documented ethnicity was selected. If two or more ethnicities had the same frequency, the most recently recorded ethnicity was chosen and prioritised in the following order: CPRD Aurum, HES APC, and HES A&E. This method helped reduce the number of missing ethnicity entries in the dataset.

Explanatory variables comprised: age (<50; 55–64; 65–74; 75–84; 85+ years); sex (male, female); cancer primary site (bone, myeloma, breast, cervix, colorectal, head and neck, liver, lung, pancreas, stomach and prostate); practice-level Index of Multiple Deprivation (IMD) measured in quintile ranging from the least [1] to most deprived [5]. IMD is a composite measure of deprivation derived from seven domains (income, employment, health and disability, education skills and training, barriers to housing and services, crime, and disorder, and living environment). We also included: practice regions (East Midlands, East of England, London, North East, North West, South Central, South West, West Midlands, Yorkshire and the Humber); practice-level urban-rural classification; palliative care status (yes or no); and number of comorbidities (0, 1, 2, 3, 4, 5+). Comorbidities were generated from a code list of 19 conditions (atrial fibrillation, coronary heart disease, heart failure, hypertension, peripheral arterial disease, stroke and transient ischaemic attack, diabetes mellitus, asthma, chronic obstructive pulmonary disease, dementia, depression, mental health, cancer (excluding primary cancer site), chronic kidney disease, epilepsy, learning disabilities, osteoporosis, rheumatoid arthritis, and non-diabetic hyperglycaemia) [42] listed on the recent UK Quality of Outcome Frameworks (QOF) [43]. Comorbidities were counted from the time of diagnosis until the time of death.

### Statistical analysis

We described the sociodemographic and clinical characteristics of patients who died between 2011 and 2021 by their ethnic groups, using counts and percentages for categorical variables, and mean (SD) and median (range) for continuous data. We calculated yearly person-time rates by dividing the aggregate monthly counts of health service use or prescription (numerator) by their respective person-months (denominator). The rates were expressed as per 1,000 person-months and stratified by ethnic groups. Person-time rates of opioid prescription and health service use over time were visualised graphically.

We developed several regression models to explore the association between ethnicity and each outcome. In the primary analyses, Poisson regression with Generalised Estimating Equations (GEE) was used to estimate adjusted rate ratios (aRRs) for [1] all opioid prescriptions, [2] multiple ED visits, and [3] multiple hospital admissions, using the five-category ethnic grouping. Each model adjusted for the same set of covariates: age, sex, cancer type, number of comorbidities, practice-level deprivation, region, urban/rural classification, palliative care status, and year of death. The comparison group for each model were patients from the White ethnicity group. Individuals with an unknown ethnicity were excluded from the main analysis. The inclusion of explanatory variables into the models was guided by previous studies [44]. We accounted for the clustering of prescribing and health service use within GP practices by including the unique GP practice identifier in each model as a nuisance parameter. The natural logarithm of the patient’s person-months at risk was included as an offset variable. The strength of the association was described using adjusted rate ratios (aRRs) and 95% confidence intervals (95% CIs.). All statistical analyses, including data management and visualisations, were completed using R Version 4.1.2 [45].

### Sensitivity analysis

We conducted sensitivity analyses to ensure the robustness of our findings. First, for the primary outcome of opioid prescription, we examined the association between opioid prescribing and patient ethnicity using a disaggregated grouping of ethnicity comprising 16 subcategories, including an “unknown group” (which included the ethnicity values of “unknown”, and patients who had missing ethnicity values), and an “other” group (for patients with an ethnicity value of “other ethnic group”) with those who are White British are regarded as the comparison patient group. Second, we conducted a stratified analysis by examining differences in the rate of prescription across various deprivation quintiles to evaluate whether the association between prescription counts and ethnicity varied across deprivation levels. Third, we calculated the quantity of opioids prescribed measured in morphine milligram equivalents (MME) to examine if there are any observable ethnic differences in the quantity of opioids prescribed in the final three months of life. The calculation of daily MME was derived by multiplying the daily dose of opioids by their equivalent analgesic value using a similar published procedure used in previous studies [46–48]. Conversion ratios and sources used for the calculation of MME are listed in the Appendix. The calculation of MME was restricted to opioids where sufficient information was available, including dosage, dose number, frequency, dose duration, and route of administration. For compound preparations such as “codeine phosphate/paracetamol” only the codeine contributed towards the calculation of MME (see Appendix for other Compound preparations). We excluded prescriptions with unreliable equianalgesic conversion ratios (e.g. sprays and suppositories) and implausible doses (e.g. prescriptions with negative dose numbers). The MME was calculated using dosage, dose number, frequency, dose duration, and route of administration. The calculation for MME considered days of drug exposure, determined by the difference between the prescription issue date and the end date or death date, whichever occurred first. To ensure that the duration of drug exposure was valid (i.e. within the 90-day window or final three months of life), the duration exceeding 90 days was rounded down to 90. We fitted a linear mixed-effect regression to examine the association between patient ethnicity and MME per person-time, adjusting for fixed effects in the main analysis and including practice identifiers as random effects. Fourth, to investigate the association between patient ethnicity and prescribing by strength of opioids, we stratified opioids into Step 2 opioids used for mild to moderate pain and Step 3 opioids used for moderate to severe pain [49]. Specifically, we developed two models, each examining the association between ethnicity and prescribing for each subset of the data. Fifth, we focused on the secondary outcomes of multiple emergency department visits and hospital admissions. Here, we developed two models to examine associations between ethnicity and these outcomes, using the 16 ethnic subcategories. All models were adjusted for the sociodemographic and clinical characteristics using the same model specification in the main analysis.

## Results

**Fig. 1:**
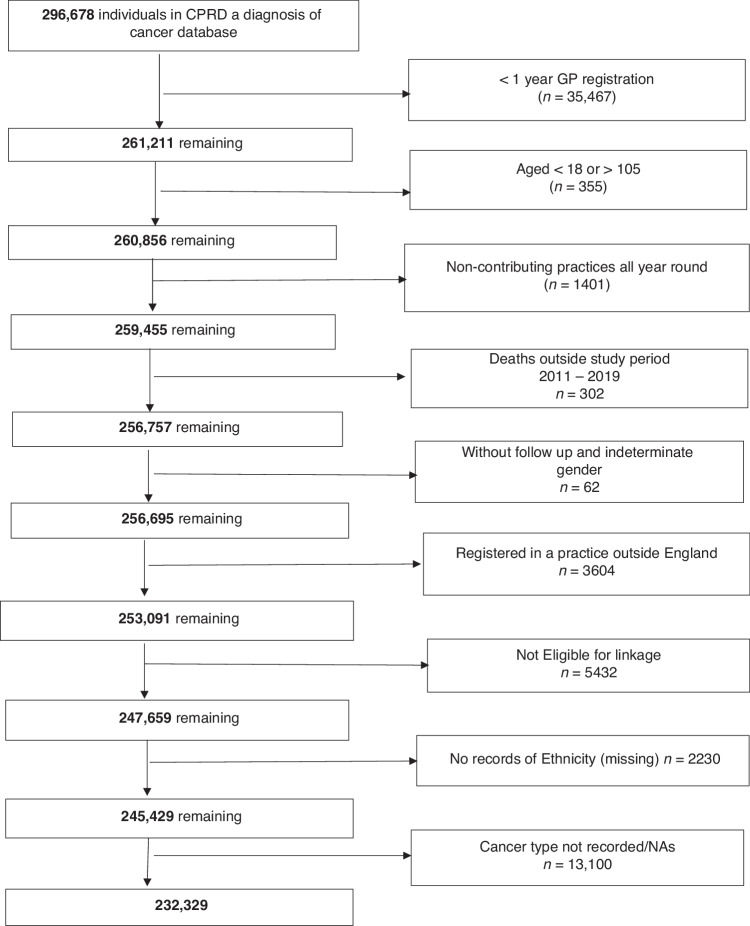
Study population.

In total, 296,678 deceased individuals were identified from the CPRD Aurum database who were diagnosed with cancer and registered with 1445 GP practices. Of these, 232,329 met our study inclusion criteria (Fig. 1).

Most cases analysed (220420, [94·9% of 232,329]) were of patients of White ethnicity. Patients from minority ethnic groups were younger (Table 1). Overall, lung cancer was the leading cause of death. However, for people of Black ethnicity, the cancer closest to death was prostate cancer (1322, [27·8% of 4752]) (Table 1).

**Table 1:** Characteristics of the study population by patient ethnic group.

Variables	Overall	Black	Mixed	Other	South Asian	White
n	232329	4752	1073	1621	4463	220420
Age (years)						
mean (SD)	77·16 (12·23)	72·36 (14·42)	71·71 (14·68)	71·19 (15·14)	71·85 (13·80)	77·44 (12·04)
Age group (%)						
18–50	6942 (3·0)	411 (8·6)	103 (9·6)	183 (11·3)	364 (8·2)	5881 (2·7)
50–59	16433 (7·1)	712 (15·0)	156 (14·5)	215 (13·3)	544 (12·2)	14806 (6·7)
60–69	38592 (16·6)	699 (14·7)	182 (17·0)	325 (20·0)	940 (21·1)	36446 (16·5)
70–79	66508 (28·6)	1210 (25·5)	286 (26·7)	371 (22·9)	1222 (27·4)	63419 (28·8)
80+	103854 (44·7)	1720 (36·2)	346 (32·2)	527 (32·5)	1393 (31·2)	99868 (45·3)
Sex (%)						
Female	108819 (46·8)	1837 (38·7)	477 (44·5)	743 (45·8)	1974 (44·2)	103788 (47·1)
Male	123510 (53·2)	2915 (61·3)	596 (55·5)	878 (54·2)	2489 (55·8)	116632 (52·9)
Practice Level Rural-Urban (%)						
Rural	33817 (14·6)	30 (0·6)	39 (3·6)	84 (5·2)	53 (1·2)	33611 (15·2)
Urban	198306 (85·4)	4719 (99·3)	1034 (96·4)	1534 (94·6)	4404 (98·7)	186615 (84·7)
Missing	206 (0·1)	3 (0·1)	0 (0·0)	3 (0·2)	6 (0·1)	194 (0·1)
Practice region (%)						
East Midlands	4312 (1·9)	68 (1·4)	8 (0·7)	17 (1·0)	64 (1·4)	4155 (1·9)
East of England	10115 (4·4)	60 (1·3)	29 (2·7)	68 (4·2)	123 (2·8)	9835 (4·5)
London	30025 (12·9)	215 (4·5)	57 (5·3)	105 (6·5)	116 (2·6)	29532 (13·4)
North East	9798 (4·2)	7 (0·1)	13 (1·2)	15 (0·9)	26 (0·6)	9737 (4·4)
North West	49773 (21·4)	238 (5·0)	106 (9·9)	191 (11·8)	474 (10·6)	48764 (22·1)
South Central	47948 (20·6)	267 (5·6)	141 (13·1)	297 (18·3)	451 (10·1)	46792 (21·2)
South West	30849 (13·3)	3371 (70·9)	562 (52·4)	760 (46·9)	2323 (52·1)	23833 (10·8)
West Midlands	40594 (17·5)	500 (10·5)	147 (13·7)	154 (9·5)	842 (18·9)	38951 (17·7)
Yorkshire & The Humber	8915 (3·8)	26 (0·5)	10 (0·9)	14 (0·9)	44 (1·0)	8821 (4·0)
Year of death (%)						
2011	19129 (8·2)	293 (6·2)	69 (6·4)	109 (6·7)	220 (4·9)	18438 (8·4)
2012	20260 (8·7)	327 (6·9)	71 (6·6)	139 (8·6)	321 (7·2)	19402 (8·8)
2013	20786 (8·9)	395 (8·3)	86 (8·0)	109 (6·7)	324 (7·3)	19872 (9·0)
2014	20045 (8·6)	365 (7·7)	94 (8·8)	117 (7·2)	345 (7·7)	19124 (8·7)
2015	20208 (8·7)	392 (8·2)	91 (8·5)	140 (8·6)	327 (7·3)	19258 (8·7)
2016	20997 (9·0)	377 (7·9)	95 (8·9)	146 (9·0)	374 (8·4)	20005 (9·1)
2017	21555 (9·3)	465 (9·8)	99 (9·2)	141 (8·7)	397 (8·9)	20453 (9·3)
2018	22072 (9·5)	468 (9·8)	104 (9·7)	157 (9·7)	469 (10·5)	20874 (9·5)
2019	22215 (9·6)	496 (10·4)	113 (10·5)	180 (11·1)	495 (11·1)	20931 (9·5)
2020	23323 (10·0)	602 (12·7)	135 (12·6)	195 (12·0)	590 (13·2)	21801 (9·9)
2021	21739 (9·4)	572 (12·0)	116 (10·8)	188 (11·6)	601 (13·5)	20262 (9·2)
Cancer primary site (%)						
Bone	3582 (1·5)	59 (1·2)	19 (1·8)	31 (1··9)	67 (1·5)	3406 (1·5)
Myeloma	5477 (2·4)	290 (6·1)	57 (5·3)	37 (2·3)	148 (3·3)	4945 (2·2)
Breast	42710 (18·4)	703 (14·8)	163 (15·2)	251 (15·5)	809 (18·1)	40784 (18·5)
Cervix	2784 (1·2)	64 (1·3)	12 (1·1)	32 (2·0)	48 (1·1)	2628 (1·2)
Colorectal	43524 (18·7)	663 (14·0)	178 (16·6)	280 (17·3)	701 (15·7)	41702 (18·9)
Head & neck	9109 (3·9)	112 (2·4)	34 (3·2)	68 (4·2)	207 (4·6)	8688 (3·9)
Liver	8974 (3·9)	196 (4·1)	51 (4·8)	116 (7·2)	348 (7·8)	8263 (3·7)
Lung	56779 (24·4)	839 (17·7)	250 (23·3)	423 (26·1)	1097 (24·6)	54170 (24·6)
Pancreas	12051 (5·2)	269 (5·7)	64 (6·0)	95 (5·9)	279 (6·3)	11344 (5·1)
Prostate	40719 (17·5)	1322 (27·8)	203 (18·9)	212 (13·1)	585 (13·1)	38397 (17·4)
Stomach	6620 (2·8)	235 (4·9)	42 (3·9)	76 (4·7)	174 (3·9)	6093 (2·8)
Palliative care quality of outcome registration (%)						
No	104799 (45·1)	2426 (51·1)	474 (44·2)	733 (45·2)	2069 (46·4)	99097 (45·0)
Yes	127530 (54·9)	2326 (48·9)	599 (55·8)	888 (54·8)	2394 (53·6)	121323 (55·0)
Number of comorbidities (%)						
0	28602 (12·3)	673 (14·2)	191 (17·8)	346 (21·3)	567 (12·7)	26825 (12·2)
1	48468 (20·9)	1026 (21·6)	190 (17·7)	413 (25·5)	797 (17·9)	46042 (20·9)
2	51734 (22·3)	1070 (22·5)	227 (21·2)	300 (18·5)	930 (20·8)	49207 (22·3)
3	43367 (18·7)	907 (19·1)	213 (19·9)	225 (13·9)	839 (18·8)	41183 (18·7)
4	29758 (12·8)	511 (10·8)	111 (10·3)	170 (10·5)	632 (14·2)	28334 (12·9)
5+	29816 (12·8)	533 (11·2)	138 (12·9)	160 (9·9)	662 (14·8)	28323 (12·8)
Missing	584 (0·3)	32 (0·7)	3 (0·3)	7 (0·4)	36 (0·8)	506 (0·2)
IMD quintile (%)						
1(Least deprived)	39288 (16·9)	175 (3·7)	132 (12·3)	252 (15·6)	406 (9·1)	38323 (17·4)
2	40359 (17·4)	269 (5·7)	107 (10·0)	219 (13·5)	539 (12·1)	39225 (17·8)
3	48632 (21·0)	873 (18·4)	207 (19·3)	365 (22·6)	831 (18·7)	46356 (21·0)
4	48475 (20·9)	1643 (34·6)	345 (32·2)	434 (26·8)	1330 (29·9)	44723 (20·3)
5 (Most deprived)	55367 (23·9)	1789 (37·7)	282 (26·3)	348 (21·5)	1349 (30·3)	51599 (23·4)

Data are *n* (%), unless otherwise specified. *IMD* Index for Multiple Deprivation.

A total of 3,987,635 opioid prescriptions were issued to 183,646 individuals (Table S1) during the study period and 620,232 of 3,987,635 prescriptions were issued to 124,639 individuals during the final three months of life (Table S2). The most common diagnosis among those receiving opioid prescriptions in the final three months of life was lung cancer, except for patients who identified as being of Black ethnicity, among whom most prescriptions were issued for patients with prostate cancer (Table S2).

The mean number of prescriptions issued in the final three months of life was higher for the patients from the White ethnic group (mean: 5·0 [SD 4·64]), compared to those patients from minority ethnic groups Black (4·43 [SD 4·30]), South Asian (4.67 [SD 4·31]), Mixed (4·37 [SD 3·86]) and Other (4.73 [SD 4·36]).

**Fig. 2:**
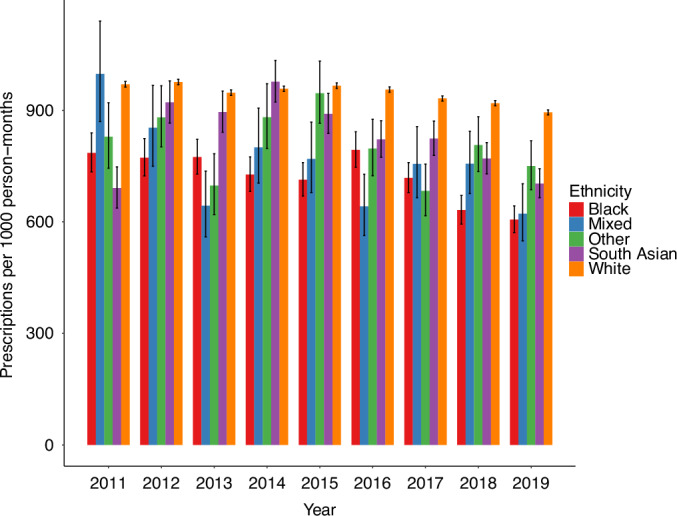
Time trends in rates of prescriptions of opioids by patient ethnicity in the final three months of life.

From 2011 to 2019 (Fig. 2), the rate of opioid prescriptions was comparatively higher for patients from the White ethnic group, varying between 969·97 and 894·43 per 1000 person-months. Overall, patients from the White group were also prescribed more Step 2 and 3 opioids (Figure S2).

**Fig. 3:**
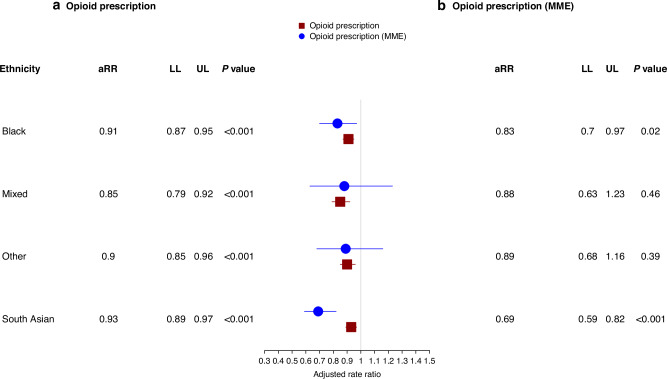
Association between ethnicity and opioid prescribing in the final three months of life. **a** Adjusted rate ratios (aRRs) with 95% confidence intervals (CIs), including lower (LL) and upper limits (UL), for the association between patient ethnicity and opioid prescriptions. **b** Adjusted rate ratios (aRRs) with 95% confidence intervals (CIs), including lower (LL) and upper limits (UL), for the association between patient ethnicity and opioid prescriptions measured in morphine milligram equivalents (MME). Patients from the White ethnic group are the reference group. An aRR value > 1 indicates a higher rate of prescription. Models were adjusted for patients’ sociodemographic characteristics, including age, cancer site, practice-level urban and rural classification, year of prescription issue, practice-level Index for Multiple Deprivation (IMD), practice region, palliative care need and number of comorbidities. Calculation of the quantity of opioid prescriptions measured in MME was restricted to opioids where sufficient information was available, including dosage, dose number, frequency, dose duration, taking into consideration the route of administration and valid days of drug supply.

**Fig. 4:**
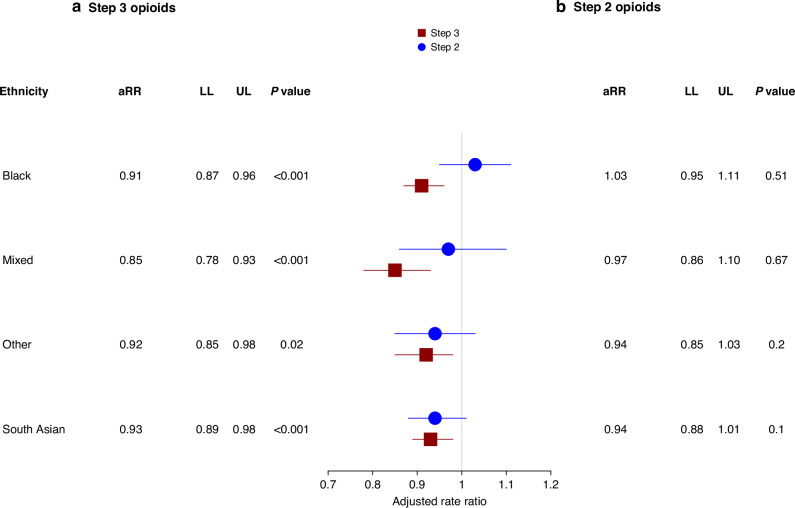
Association between ethnicity and prescription of Step 3 and Step 2 opioids in the final three months of life. **a** Adjusted rate ratios (aRRs) with 95% confidence intervals (CIs), including lower (LL) and upper limits (UL), for the association between patient ethnicity and prescription of Step 3 opioids. **b** Adjusted rate ratios (aRRs) with 95% confidence intervals (CIs), including lower (LL) and upper limits (UL), for the association between patient ethnicity and prescription of Step 2 opioids. Patients from the White ethnic group are the comparison group. An aRR value > 1 indicates a higher rate of prescription. Models were adjusted for patients’ sociodemographic characteristics, including age, cancer type, practice-level urban and rural classification, year of prescription issue, practice-level Index for Multiple Deprivation (IMD), practice region, palliative care need and number of comorbidities.

After adjustment for sociodemographic and clinical characteristics (Fig. 3a), the aRRs of opioid prescribing remained lower for patients from all minority ethnic groups (Black 0·91 [95% CI: 0·87–0·95], South Asian 0·93 [0·89–0·97], Mixed 0·85 [0·79–0·92] and Other 0·90 [0·85–0·96]) than those from the White ethnic group. These disparities were not only observed in the likelihood of receiving an opioid prescription, but also in the quantity (in MME) and strength of opioids prescribed (Step 2 and Step 3 opioids). Specifically, the results of sensitivity analyses based on the quantity of opioids (Fig. 3b) and strength (Fig. 4a, b) were consistent with the results of the main analysis. In terms of quantity, patients from all minority ethnic groups were prescribed lower rates of opioids (Black 0·83 [95% CI: 0·70–0.97], South Asian 0·69 [0·59– 0·82], Mixed: 0·88 [0·63–1.23], Other ethnic groups 0·89 [0·68–1·16]) compared to patients from the White ethnic group. Similarly, patients from minority ethnic backgrounds had a lower rate of Step 2 or Step 3 opioids, except patients from the Black ethnic group, who were prescribed more Step 2 opioids (1·03 [0·95–1·11]). The results of sensitivity analysis based on 16 ethnic subcategories (Table S3, Figure S3) and stratification by deprivation quintiles did not differ significantly from those of the main analysis (Table S6).

**Fig. 5:**
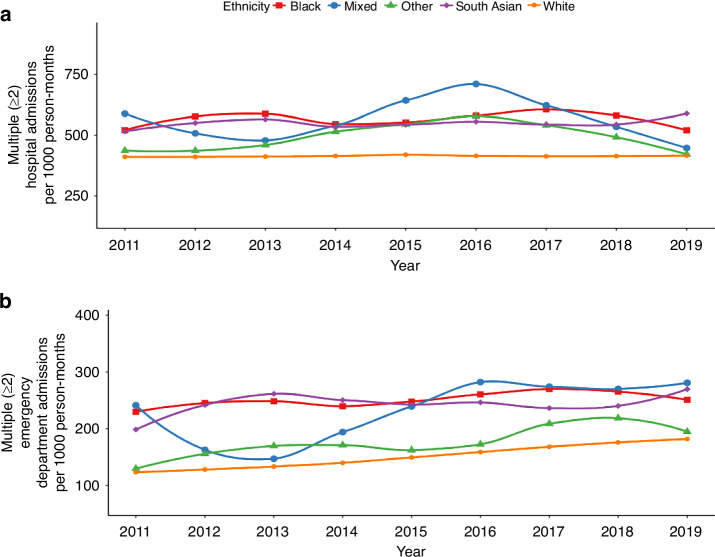
Time trends in multiple hospital admissions and emergency department visits by patient ethnicity. **a** Rates of multiple hospital admissions over time. **b** Rates of multiple emergency department visits over time.

**Fig. 6:**
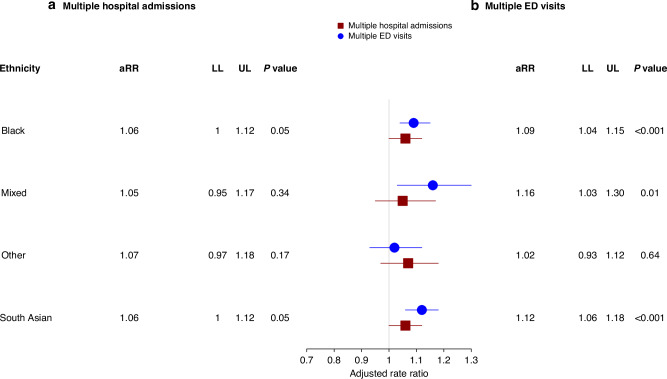
Association between ethnicity and health service use in the final three months of life. **a** Adjusted rate ratios (aRRs) with 95% confidence intervals (CIs), including lower (LL) and upper limits (UL), for the association between patient ethnicity and multiple hospital admissions. **b** Adjusted rate ratios (aRRs) with 95% confidence intervals (CIs), including lower (LL) and upper limits (UL), for the association between patient ethnicity and multiple emergency department visits in the final three months of life. Patients from the White ethnic group are the comparison group. An aRR value > 1 indicates a higher rate of service use than the comparison category. Models were adjusted for patients’ sociodemographic characteristics, including age, cancer type, Practice-Level urban-rural classification, year of prescription issue, practice-level Index for Multiple Deprivation (IMD), practice region, palliative care need and number of comorbidities.

A total of 2,314,076 hospital admissions occurred during the study period, where 278,510 [12.0% of 2,314,076] occurred during the final three months of life (Table S4). The rates of multiple hospital admissions varied between the ethnic groups (Fig. 5a). Compared to patients from the White ethnic group, the rate of multiple hospital admissions (ranging between 406.8 and 420 per 1000 person-months) was higher among patients from minority ethnic groups. After adjustment for sociodemographic and clinical characteristics (Fig. 6a), compared to patients from the White ethnic group, patients from minority ethnic backgrounds had higher rate of multiple hospital admissions (Black 1·06 [95%CI: 1·00–1·12], South Asian 1·06 [1·00–1·12], Mixed 1.05 [0·95–1·17] and Other 1·07 [0·97–1·18]). The results of sensitivity analysis based on a cohort of individuals in the final six months of life (Figure S4B) and the 16 ethnic subgroups in the final three months of life (Figure S5A) also showed a similar increase in the rate of hospital admissions among people from minority ethnic backgrounds.

A total of 786,143 ED visits occurred during the study period, where 103,165 [7·1% of 786,143] related to ED visits in the final three months of life (Table S5). The rates of multiple ED visits were comparatively higher for patients from minority ethnic backgrounds (ranging between 122.6 and 181.1 per 1000 person-months) compared with individuals from the White ethnic group (Fig. 5b). After adjustment for sociodemographic and clinical characteristics, the rates of multiple ED visits (Fig. 6b) remained higher for patients from minority ethnic groups (Black 1·09 [95%CI: 1·04–1·15], South Asian 1·12 [1·06–1·18], Mixed 1·16 [1·03–1·30] and Other 1·02 [0·93–1·12]). The results of sensitivity analysis based on a cohort of individuals in the final six months of life (Figure S4A) were also similar the results of the main analysis. The aRRs based on the 16 ethnic subgroups showed increased multiple ED visits for patients from minority ethnic subgroups, compared to patients from the White ethnic group (Figure S5B)

## Discussion

To our knowledge, this is the first population-based study in England that has applied an intersectional lens to examine the association between patient ethnicity and opioid prescribing for cancer pain in the last three months of life. Specifically, our findings show that individuals from minority ethnic backgrounds were less likely to receive a prescription for opioids compared to those from the White ethnic group. Furthermore, among those who did receive opioids, the quantity prescribed, measured in morphine milligram equivalents (MME), was less than that prescribed to those from the White ethnic group.

These results highlight inequities both in terms of access to opioid prescriptions and the dosing of opioids at the end of life. We also observed higher rates of multiple ED visits and hospital admissions among patients from minority ethnic backgrounds. These disparities persisted even after adjusting for sociodemographic characteristics, including age, sex, comorbidity, cancer site, palliative care needs, place of death, practice region, socioeconomic status, and urban-rural practice level. These findings align with international studies [11, 50], but must be interpreted cautiously.

The underlying causes for these disparities remain unclear, potentially involving patient, professional, organisational, and system-centred factors. For example, ethnic differences in preferences for opioids might be associated with the results. Some individuals’ religious or cultural beliefs may impact their response to illness and symptoms [51]. Fears of dependency and side effects [52, 53], along with potential underreporting of pain due to the perceived social status of physicians, may also play a role [54]. Language barriers may further challenge the communication of pain history [55]. The COVID-19 pandemic highlighted ethnic differences in healthcare experiences and outcomes and mistrust in medical advice [56, 57]. Clinician and health system characteristics also affect how preferences are elicited and addressed. In the USA, clinicians may be reluctant to prescribe opioids to patients from minority ethnic backgrounds due to perceived risks of misuse and addiction [58]. This might explain the higher rates of Step 2 opioids, associated with managing weak to moderate pain, rather than Step 3 opioids, principally used for moderate to severe pain for some patients from minority ethnic backgrounds. Bias among physicians, whether conscious or unconscious, can lead to undertreatment when patients’ pain severity assessments differ from their own [59, 60]. To what extent this issue is present in clinical encounters between clinicians and patients from minority ethnic backgrounds in England has not been examined.

We also examined the association between patient ethnicity and ED visits and hospital admissions in the last three months of life. Although causation cannot be inferred, patients from minority ethnic backgrounds had a higher rate of multiple ED visits and hospital admissions compared to patients from the White ethnic group. These differences persisted over time and after adjusting for sociodemographic and clinical characteristics. Previous UK research has reported similar trends, with higher ED visits [19, 61] and hospital admissions among patients from Asian and Black minority ethnic backgrounds [20]. Understanding factors contributing to these visits and admissions, such as poorly managed pain, may help healthcare professionals provide better support.

This study is associated with the following limitations. The CPRD records only GP prescriptions, potentially underestimating true opioid prescribing levels due to exclusions from oncology and palliative care services. GP prescriptions, however, account for the majority of NHS opioid prescription costs [30]. Future research could, nevertheless, incorporate hospital prescribing data, where available, to better quantify total opioid use, including inpatient initiation or titration, which is not captured in our current dataset. Measuring opioid exposure using prescription records is complex due to the non-capture of unfilled prescriptions, patient administration, and as-needed medication use. The lack of patient-level pain scores, disease severity also limits the study. The CPRD database includes only participating GP practices, possibly representing better practices [27]. We used a limited subset of drugs to calculate morphine equivalents, with potential inaccuracies in conversion values. Future studies could calculate morphine equivalence with a larger dataset by using national averages instead of individual-level data. The use of practice-level deprivation indices as socioeconomic proxies risks ecological fallacy [62]. While we examined multiple hospital admissions, not all are associated with poor care experiences. Comorbidity was measured as a count of the number of comorbidities associated with pain; a weighted score may improve validity [63]. Whilst we categorised individuals into ethnic groupings is in keeping with other studies [39], it could be construed as being overly reductionist [64]. Nevertheless, the results of the sensitivity analyses using the more granular 16 ethnic subcategories did not differ from the five major ethnic groups. During the study period, UK NICE standards for palliative and end-of-life care [65] and the Lancet Oncology Commission [66] emphasised the importance of effective pain management and cautioned against overgeneralising fears about opioid misuse from the United States to cancer pain globally. Nevertheless, broader policy concerns about opioid misuse, primarily in non-cancer contexts [67] may have contributed to a more cautious prescribing climate in general practice. While this shift does not fully explain the ethnic disparities observed, it may have compounded existing inequities if caution was applied unevenly across patient groups. As with other as studies [19, 68], we lacked data on both the reasons for emergency department visits and opioid prescriptions issued directly during ED visits or hospital stays. It is therefore not possible to determine whether increased ED visits among patients from minority ethnic backgrounds reflect unmet pain needs, preferences for accessing care via ED rather than general practice, or other factors such as difficulties accessing primary care. The absence of hospital/ED prescribing data may partially contribute to observed disparities in community prescribing, particularly if some patients rely on these settings as their primary source of pain relief. Understanding whether ED visits reflect preferred access, unmet needs, or structural barriers would require a prospective study.

This study analysed data from over 232,000 individuals in a nine-year cohort. Reports from three Lancet Commissions [10] [66, 69] have emphasised the need to tackle inequalities in pain treatment and improve end-of-life care. Despite ongoing NHS and cancer charity initiatives, significant ethnic disparities in opioid prescribing persist and are associated with higher emergency department visits and hospital admissions in the final three months of life. Our findings confirm ongoing inequities in end-of-life care among patients from minority ethnic communities in the UK and internationally [11, 25, 61, 64]. We show that patient ethnicity is significantly associated with opioid prescribing patterns for managing cancer pain at the end of life. Ignoring this issue is ethically indefensible and compounds health inequities, undermining fairness and cultural safety. Addressing these disparities will require coordinated policy, clinical, and research efforts to ensure all patients with advanced cancer receive equitable and appropriate pain relief in their final months of life.

## Supplementary information

Supplementary Tables and Figures

Strobe template form

Appendix_Dec_02_2022_cancercodes.txt

## Data Availability

Complete codes are provided online.
