# Supplementary material for: Ethnic disparities in opioid prescribing for cancer pain and associated emergency department visits and hospital admissions in the last three months of life: a retrospective cohort study
Source: Br J Cancer. 2025 Sep 25;133(11):1675–85. doi: 10.1038/s41416-025-03200-4 (PMC12644774; doi:10.1038/s41416-025-03200-4)
Supplement: Supplementary file 1 — Supplementary Tables and Figures [file 41416_2025_3200_MOESM1_ESM.docx]

**Ethnic disparities in opioid prescribing for cancer pain and associated emergency department visits and hospital admissions in the last three months of life: a retrospective cohort study**

**Supplementary: Tables and Figures:**

Appendix 1. List of opioids and conversion table

Appendix 2. Opioid conversion source link

Table S1: Characteristics of individuals who were prescribed opioids by ethnic group

Table S2: Characteristics of individuals who were prescribed opioids by ethnic group in the final three months of life

Table S3: Association between Patient Ethnicity and Opioid Prescribing (16 Ethnic subgroups)

Table S4: Characteristics of individuals who were admitted to the hospital by ethnic group between 2011 to 2019

Table S5: Characteristics of individuals who visited ED by ethnic group between 2011 to 2019

Figure S1: Trends in the rate of Opioid prescription by ethnic group by ethnic group between 2011 to 2019·

Figure S2: Trends in the rate of prescription of Step 2 & Step 3 Opioids by ethnic group between 2011 to 2019·

Figure S3: Forest plot of the association between Patient Ethnicity and Step 3 & 2 Opioids - Final three months of life (16 ethnic subcategories)

Figure S4: Forest plot of the association between health service use and Patient Ethnicity in the last 6 months of life

Figure S5**:** Forest plot of the association between Health service use and Patient Ethnicity (16 ethnic subgroups) – Final three months of life:

Table S6: adjusted rate ratio (aRRs) of the association between patient ethnicity and opioid prescriptions in the final three months of life (stratified analysis by deprivation quintiles)

**Appendix:**

**Appendix 1.** List of opioids and conversion table:

| **Drug Name** | **Term from EMIS** | **Opioid dose (mg)** | | **Conversion factor** | **OMEQ** | **Source** |
| --- | --- | --- | --- | --- | --- | --- |
| alfentanil | alfentanil 1mg/2ml solution for injection ampoules | 0.5 | | 30 | 15 | PCGP |
| alfentanil | alfentanil 5mg/10ml solution for injection ampoules | 0.5 | | 30 | 15 | PCGP |
| alfentanil | alfentanil 5mg/1ml solution for injection ampoules | 5 | | 30 | 150 | PCGP |
| alfentanil | alfentanil injection 500 micrograms/ml | 0.5 | | 30 | 15 | PCGP |
| alfentanil | rapifen 1mg/2ml solution for injection ampoules (piramal critical care ltd) | 0.5 | | 30 | 15 | PCGP |
| alfentanil | rapifen 5mg/10ml solution for injection ampoules (piramal critical care ltd) | 0.5 | | 30 | 15 | PCGP |
| alfentanil | rapifen injection 500 micrograms/ml | 0.5 | | 30 | 15 | PCGP |
| alfentanil | rapifen intensive care 5mg/1ml solution for injection ampoules (piramal critical care ltd) | 5 | | 30 | 150 | PCGP |
| aspirin/codeine | Aspirin 500mg / Codeine 8mg dispersible tablets sugar-free | 8 | | 0.1 | 0.8 | PCGP |
| aspirin/codeine | Co-codaprin 8mg/400mg dispersible tablets | 8 | | 0.1 | 0.8 | PCGP |
| aspirin/codeine | Co-codaprin 8mg/400mg tablets | 8 | | 0.1 | 0.8 | PCGP |
| buclizine/codeine/paracetamol | Migraleve Pink tablets (McNeil Products Ltd) | 8 | | 0.1 | 0.8 | PCGP |
| buprenorphine | bunov 10 micrograms/hour transdermal patches (glenmark pharmaceuticals europe ltd) | 0.24 | | 100 | 24 | PCGP |
| buprenorphine | bunov 20 micrograms/hour transdermal patches (glenmark pharmaceuticals europe ltd) | 0.48 | | 100 | 48 | PCGP |
| buprenorphine | bunov 5 micrograms/hour transdermal patches (glenmark pharmaceuticals europe ltd) | 0.12 | | 100 | 12 | PCGP |
| buprenorphine | bupeaze 35 micrograms/hour transdermal patches (dr reddy's laboratories (uk) ltd) | 0.84 | | 100 | 84 | PCGP |
| buprenorphine | bupeaze 52.5 micrograms/hour transdermal patches (dr reddy's laboratories (uk) ltd) | 1.26 | | 100 | 126 | PCGP |
| buprenorphine | bupeaze 70micrograms/hour transdermal patches (dr reddy's laboratories (uk) ltd) | 1.68 | | 100 | 168 | PCGP |
| buprenorphine | buplast 35micrograms/hour transdermal patches (mylan) | 0.84 | | 100 | 84 | PCGP |
| buprenorphine | buplast 52.5micrograms/hour transdermal patches (mylan) | 1.26 | | 100 | 126 | PCGP |
| buprenorphine | buplast 70micrograms/hour transdermal patches (mylan) | 1.68 | | 100 | 168 | PCGP |
| buprenorphine | bupramyl 10micrograms/hour transdermal patches (mylan) | 0.24 | | 100 | 24 | PCGP |
| buprenorphine | bupramyl 20micrograms/hour transdermal patches (mylan) | 0.48 | | 100 | 48 | PCGP |
| buprenorphine | bupramyl 5micrograms/hour transdermal patches (mylan) | 0.12 | | 100 | 12 | PCGP |
| buprenorphine | buprenorphine 10micrograms/hour transdermal patches | 0.24 | | 100 | 24 | PCGP |
| buprenorphine | buprenorphine 128mg/0.36ml prolonged-release solution for injection pre-filled syringes | 355 | | 100 | 35500 | UCSF |
| buprenorphine | buprenorphine 15micrograms/hour transdermal patches | 0.36 | | 100 | 36 | PCGP |
| buprenorphine | buprenorphine 16mg/0.32ml prolonged-release solution for injection pre-filled syringes | 50 | | 100 | 5000 | UCSF |
| buprenorphine | buprenorphine 20micrograms/hour transdermal patches | 0.48 | | 100 | 48 | PCGP |
| buprenorphine | buprenorphine 24mg/0.48ml prolonged-release solution for injection pre-filled syringes | 50 | | 100 | 5000 | UCSF |
| buprenorphine | buprenorphine 32mg/0.64ml prolonged-release solution for injection pre-filled syringes | 50 | | 100 | 5000 | UCSF |
| buprenorphine | buprenorphine 35micrograms/hour transdermal patches | 0.84 | | 100 | 84 | PCGP |
| buprenorphine | buprenorphine 52.5micrograms/hour transdermal patches | 1.26 | | 100 | 126 | PCGP |
| buprenorphine | buprenorphine 5micrograms/hour transdermal patches | 0.12 | | 100 | 12 | PCGP |
| buprenorphine | buprenorphine 64mg/0.18ml prolonged-release solution for injection pre-filled syringes | 355 | | 100 | 35500 | UCSF |
| buprenorphine | buprenorphine 70micrograms/hour transdermal patches | 1.68 | | 100 | 168 | PCGP |
| buprenorphine | buprenorphine 8mg/0.16ml prolonged-release solution for injection pre-filled syringes | 50 | | 100 | 5000 | UCSF |
| buprenorphine | buprenorphine 96mg/0.27ml prolonged-release solution for injection pre-filled syringes | 355 | | 100 | 35500 | UCSF |
| buprenorphine | buprenorphine injection 300 micrograms | 0.3 | | 100 | 30 | UCSF |
| buprenorphine | busiete 10micrograms/hour transdermal patches (teva uk ltd) | 0.24 | | 100 | 24 | PCGP |
| buprenorphine | busiete 20micrograms/hour transdermal patches (teva uk ltd) | 0.48 | | 100 | 48 | PCGP |
| buprenorphine | busiete 5micrograms/hour transdermal patches (teva uk ltd) | 0.12 | | 100 | 12 | PCGP |
| buprenorphine | butec 10micrograms/hour transdermal patches (qdem pharmaceuticals ltd) | 0.24 | | 100 | 24 | PCGP |
| buprenorphine | butec 15micrograms/hour transdermal patches (qdem pharmaceuticals ltd) | 0.36 | | 100 | 36 | PCGP |
| buprenorphine | butec 20micrograms/hour transdermal patches (qdem pharmaceuticals ltd) | 0.48 | | 100 | 48 | PCGP |
| buprenorphine | butec 5micrograms/hour transdermal patches (qdem pharmaceuticals ltd) | 0.12 | | 100 | 12 | PCGP |
| buprenorphine | butrans 10micrograms/hour transdermal patches (napp pharmaceuticals ltd) | 0.24 | | 100 | 24 | PCGP |
| buprenorphine | butrans 15micrograms/hour transdermal patches (napp pharmaceuticals ltd) | 0.36 | | 100 | 36 | PCGP |
| buprenorphine | butrans 20micrograms/hour transdermal patches (napp pharmaceuticals ltd) | 0.48 | | 100 | 48 | PCGP |
| buprenorphine | butrans 5micrograms/hour transdermal patches (napp pharmaceuticals ltd) | 0.12 | | 100 | 12 | PCGP |
| buprenorphine | buvidal 128mg/0.36ml prolonged-release solution for injection pre-filled syringes (camurus ab) | 355 | | 100 | 35500 | UCSF |
| buprenorphine | buvidal 16mg/0.32ml prolonged-release solution for injection pre-filled syringes (camurus ab) | 50 | | 100 | 5000 | UCSF |
| buprenorphine | buvidal 24mg/0.48ml prolonged-release solution for injection pre-filled syringes (camurus ab) | 50 | | 100 | 5000 | UCSF |
| buprenorphine | buvidal 32mg/0.64ml prolonged-release solution for injection pre-filled syringes (camurus ab) | 50 | | 100 | 5000 | UCSF |
| buprenorphine | buvidal 64mg/0.18ml prolonged-release solution for injection pre-filled syringes (camurus ab) | 355 | | 100 | 35500 | UCSF |
| buprenorphine | buvidal 8mg/0.16ml prolonged-release solution for injection pre-filled syringes (camurus ab) | 50 | | 100 | 5000 | UCSF |
| buprenorphine | buvidal 96mg/0.27ml prolonged-release solution for injection pre-filled syringes (camurus ab) | 355 | | 100 | 35500 | UCSF |
| buprenorphine | carlosafine 35micrograms/hour transdermal patches (glenmark pharmaceuticals europe ltd) | 0.84 | | 100 | 84 | PCGP |
| buprenorphine | carlosafine 52.5micrograms/hour transdermal patches (glenmark pharmaceuticals europe ltd) | 1.26 | | 100 | 126 | PCGP |
| buprenorphine | carlosafine 70micrograms/hour transdermal patches (glenmark pharmaceuticals europe ltd) | 1.68 | | 100 | 168 | PCGP |
| buprenorphine | hapoctasin 35micrograms/hour transdermal patches (accord healthcare ltd) | 0.84 | | 100 | 84 | PCGP |
| buprenorphine | hapoctasin 52.5micrograms/hour transdermal patches (accord healthcare ltd) | 1.26 | | 100 | 126 | PCGP |
| buprenorphine | hapoctasin 70micrograms/hour transdermal patches (accord healthcare ltd) | 1.68 | | 100 | 168 | PCGP |
| buprenorphine | panitaz 10micrograms/hour transdermal patches (dr reddy's laboratories (uk) ltd) | 0.24 | | 100 | 24 | PCGP |
| buprenorphine | panitaz 20micrograms/hour transdermal patches (dr reddy's laboratories (uk) ltd) | 0.48 | | 100 | 48 | PCGP |
| buprenorphine | panitaz 5micrograms/hour transdermal patches (dr reddy's laboratories (uk) ltd) | | 0.12 | 100 | 12 | PCGP |
| buprenorphine | prenotrix 35micrograms/hour transdermal patches (genesis pharmaceuticals ltd) | | 0.84 | 100 | 84 | PCGP |
| buprenorphine | prenotrix 52.5micrograms/hour transdermal patches (genesis pharmaceuticals ltd) | | 1.26 | 100 | 126 | PCGP |
| buprenorphine | prenotrix 70micrograms/hour transdermal patches (genesis pharmaceuticals ltd) | | 1.68 | 100 | 168 | PCGP |
| buprenorphine | reletrans 10micrograms/hour transdermal patches (sandoz ltd) | | 0.24 | 100 | 24 | PCGP |
| buprenorphine | reletrans 15micrograms/hour transdermal patches (sandoz ltd) | | 0.36 | 100 | 36 | PCGP |
| buprenorphine | reletrans 20micrograms/hour transdermal patches (sandoz ltd) | | 0.48 | 100 | 48 | PCGP |
| buprenorphine | reletrans 5micrograms/hour transdermal patches (sandoz ltd) | | 0.12 | 100 | 12 | PCGP |
| buprenorphine | relevtec 35micrograms/hour transdermal patches (sandoz ltd) | | 0.84 | 100 | 84 | PCGP |
| buprenorphine | relevtec 52.5micrograms/hour transdermal patches (sandoz ltd) | | 1.26 | 100 | 126 | PCGP |
| buprenorphine | relevtec 70micrograms/hour transdermal patches (sandoz ltd) | | 1.68 | 100 | 168 | PCGP |
| buprenorphine | sevodyne 10micrograms/hour transdermal patches (aspire pharma ltd) | | 0.24 | 100 | 24 | PCGP |
| buprenorphine | sevodyne 15micrograms/hour transdermal patches (aspire pharma ltd) | | 0.36 | 100 | 36 | PCGP |
| buprenorphine | sevodyne 20micrograms/hour transdermal patches (aspire pharma ltd) | | 0.48 | 100 | 48 | PCGP |
| buprenorphine | sevodyne 5micrograms/hour transdermal patches (aspire pharma ltd) | | 0.12 | 100 | 12 | PCGP |
| buprenorphine | transtec 35micrograms/hour transdermal patches (napp pharmaceuticals ltd) | | 0.84 | 100 | 84 | PCGP |
| buprenorphine | transtec 52.5micrograms/hour transdermal patches (napp pharmaceuticals ltd) | | 1.26 | 100 | 126 | PCGP |
| buprenorphine | transtec 70micrograms/hour transdermal patches (napp pharmaceuticals ltd) | | 1.68 | 100 | 168 | PCGP |
| buprenorphine | turgeon 35micrograms/hour transdermal patches (teva uk ltd) | | 0.84 | 100 | 84 | PCGP |
| buprenorphine | turgeon 52.5micrograms/hour transdermal patches (teva uk ltd) | | 1.26 | 100 | 126 | PCGP |
| buprenorphine | turgeon 70micrograms/hour transdermal patches (teva uk ltd) | | 1.68 | 100 | 168 | PCGP |
| buprenorphine | buprenorphine 1mg sublingual tablets sugar free | | 1 | 60 | 60 | PCGP |
| buprenorphine | buprenorphine 200microgram sublingual tablets sugar free | | 0.2 | 60 | 12 | PCGP |
| buprenorphine | buprenorphine 2mg oral lyophilisates sugar free | | 2 | 60 | 120 | OD |
| buprenorphine | buprenorphine 2mg sublingual tablets sugar free | | 2 | 60 | 120 | PCGP |
| buprenorphine | buprenorphine 300micrograms/1ml solution for injection ampoules | | 0.3 | 100 | 30 | UCSF |
| buprenorphine | buprenorphine 400microgram sublingual tablets sugar free | | 0.4 | 60 | 24 | PCGP |
| buprenorphine | buprenorphine 4mg sublingual tablets sugar free | | 4 | 60 | 240 | PCGP |
| buprenorphine | buprenorphine 6mg sublingual tablets sugar free | | 6 | 60 | 360 | PCGP |
| buprenorphine | buprenorphine 8mg oral lyophilisates sugar free | | 8 | 60 | 480 | OD |
| buprenorphine | buprenorphine 8mg sublingual tablets sugar free | | 8 | 60 | 480 | PCGP |
| buprenorphine | espranor 2mg oral lyophilisates (martindale pharmaceuticals ltd) | | 2 | 60 | 120 | OD |
| buprenorphine | espranor 8mg oral lyophilisates (martindale pharmaceuticals ltd) | | 8 | 60 | 480 | OD |
| buprenorphine | gabup 0.4mg sublingual tablets (martindale pharmaceuticals ltd) | | 0.4 | 60 | 24 | PCGP |
| buprenorphine | gabup 1mg sublingual tablets (martindale pharmaceuticals ltd) | | 1 | 60 | 60 | PCGP |
| buprenorphine | gabup 2mg sublingual tablets (martindale pharmaceuticals ltd) | | 2 | 60 | 120 | PCGP |
| buprenorphine | gabup 4mg sublingual tablets (martindale pharmaceuticals ltd) | | 4 | 60 | 240 | PCGP |
| buprenorphine | gabup 6mg sublingual tablets (martindale pharmaceuticals ltd) | | 6 | 60 | 360 | PCGP |
| buprenorphine | gabup 8mg sublingual tablets (martindale pharmaceuticals ltd) | | 8 | 60 | 480 | PCGP |
| buprenorphine | prefibin 0.4mg sublingual tablets (sandoz ltd) | | 0.4 | 60 | 24 | PCGP |
| buprenorphine | prefibin 2mg sublingual tablets (sandoz ltd) | | 2 | 60 | 120 | PCGP |
| buprenorphine | prefibin 8mg sublingual tablets (sandoz ltd) | | 8 | 60 | 480 | PCGP |
| buprenorphine | subutex 0.4mg sublingual tablets (indivior uk ltd) | | 0.4 | 60 | 24 | PCGP |
| buprenorphine | subutex 2mg sublingual tablets (indivior uk ltd) | | 2 | 60 | 120 | PCGP |
| buprenorphine | subutex 8mg sublingual tablets (indivior uk ltd) | | 8 | 60 | 480 | PCGP |
| buprenorphine | temgesic 200microgram sublingual tablets (indivior uk ltd) | | 0.2 | 60 | 12 | PCGP |
| buprenorphine | temgesic 300micrograms/1ml solution for injection ampoules (indivior uk ltd) | | 0.3 | 100 | 30 | UCSF |
| buprenorphine | temgesic 400microgram sublingual tablets (indivior uk ltd) | | 0.4 | 60 | 24 | PCGP |
| buprenorphine | temgesic injection 300 micrograms | | 0.3 | 100 | 30 | UCSF |
| buprenorphine | tephine 200microgram sublingual tablets (sandoz ltd) | | 0.2 | 60 | 12 | PCGP |
| buprenorphine | tephine 400microgram sublingual tablets (sandoz ltd) | | 0.4 | 60 | 24 | PCGP |
| buprenorphine/naloxone | buprenorphine 16mg / naloxone 4mg sublingual tablets sugar free | | 16 | 60 | 960 | PCGP |
| buprenorphine/naloxone | buprenorphine 2mg / naloxone 500microgram sublingual tablets sugar free | | 2 | 60 | 120 | PCGP |
| buprenorphine/naloxone | buprenorphine 8mg / naloxone 2mg sublingual tablets sugar free | | 8 | 60 | 480 | PCGP |
| buprenorphine/naloxone | Suboxone 2mg/500microgram sublingual tablets (Indivior UK Ltd) | | 2 | 60 | 120 | PCGP |
| buprenorphine/naloxone | Suboxone 8mg/2mg sublingual tablets (Indivior UK Ltd) | | 8 | 60 | 480 | PCGP |
| caffeine/codeine/doxylamine/paracetamol | Syndol caplets (Sanofi) | | 10 | 0.1 | 1 | PCGP |
| caffeine/codeine/paracetamol | Solpadeine Max soluble tablets (Omega Pharma Ltd) | | 12.8 | 0.1 | 1.28 | PCGP |
| caffeine/codeine/paracetamol | Solpadeine Plus capsules (Omega Pharma Ltd) | | 8 | 0.1 | 0.8 | PCGP |
| caffeine/codeine/paracetamol | Solpadeine Plus soluble tablets (Omega Pharma Ltd) | | 8 | 0.1 | 0.8 | PCGP |
| chloroform/kaolin/morphine/sodium | Kaolin and Morphine mixture | |  | 1 | 0 | PCGP |
| cocaine/ethanol/morphine | morphine and cocaine elixir | |  | 1 | 0 | PCGP |
| codeine | codeine 15mg tablets | | 15 | 0.1 | 1.5 | PCGP |
| codeine | codeine 15mg/5ml linctus | | 3 | 0.1 | 0.3 | PCGP |
| codeine | codeine 15mg/5ml linctus sugar free | | 3 | 0.1 | 0.3 | PCGP |
| codeine | codeine 25mg/5ml oral solution | | 5 | 0.1 | 0.5 | PCGP |
| codeine | codeine 30mg tablets | | 30 | 0.1 | 3 | PCGP |
| codeine | codeine 3mg/5ml linctus paediatric | | 0.6 | 0.1 | 0.06 | PCGP |
| codeine | codeine 60mg tablets | | 60 | 0.1 | 6 | PCGP |
| codeine | codeine 60mg/1ml solution for injection ampoules | | 60 | 0.3 | 18 | UCSF |
| codeine | codeine linctus diabetic linctus 15 mg/5 ml | | 3 | 0.1 | 0.3 | PCGP |
| codeine | codeine phosphate linctus 15 mg/5 ml | | 3 | 0.1 | 0.3 | PCGP |
| codeine | codeine phosphate powder | | 1 | 0.1 | 0.1 | PCGP |
| dihydrocodeine | dihydrocodeine tartrate elixir 10mg/5 ml | | 2 | 0.1 | 0.2 | PCGP |
| dihydrocodeine | dihydrocodeine tartrate tablets 30 mg | | 30 | 0.1 | 3 | PCGP |
| codeine | galcodine 15mg/5ml linctus (thornton & ross ltd) | | 3 | 0.1 | 0.3 | PCGP |
| codeine | galcodine 3mg/5ml linctus paediatric (thornton & ross ltd) | | 0.6 | 0.1 | 0.06 | PCGP |
| codeine/ibuprofen | Ibuprofen 200mg / Codeine 12.8mg tablets | | 12.8 | 0.1 | 1.28 | PCGP |
| codeine/ibuprofen | Nurofen Plus tablets (Reckitt Benckiser Healthcare (UK) Ltd) | | 12.8 | 0.1 | 1.28 | PCGP |
| codeine/paracetamol | Co-codamol 12.8mg/500mg tablets | | 12.8 | 0.1 | 1.28 | PCGP |
| codeine/paracetamol | Co-codamol 15mg/500mg capsules | | 15 | 0.1 | 1.5 | PCGP |
| codeine/paracetamol | Co-codamol 15mg/500mg effervescent tablets sugar free | | 15 | 0.1 | 1.5 | PCGP |
| codeine/paracetamol | Co-codamol 15mg/500mg tablets | | 15 | 0.1 | 1.5 | PCGP |
| codeine/paracetamol | Co-codamol 30mg/500mg capsules | | 30 | 0.1 | 3 | PCGP |
| codeine/paracetamol | Co-codamol 30mg/500mg effervescent tablets | | 30 | 0.1 | 3 | PCGP |
| codeine/paracetamol | Co-codamol 30mg/500mg tablets | | 30 | 0.1 | 3 | PCGP |
| codeine/paracetamol | Co-codamol 8mg/500mg caplets (Vantage) | | 8 | 0.1 | 0.8 | PCGP |
| codeine/paracetamol | Co-codamol 8mg/500mg capsules | | 8 | 0.1 | 0.8 | PCGP |
| codeine/paracetamol | Co-codamol 8mg/500mg effervescent tablets | | 8 | 0.1 | 0.8 | PCGP |
| codeine/paracetamol | Co-codamol 8mg/500mg tablets | | 8 | 0.1 | 0.8 | PCGP |
| codeine/paracetamol | Codipar 15mg/500mg capsules (Advanz Pharma) | | 15 | 0.1 | 1.5 | PCGP |
| codeine/paracetamol | Codipar 15mg/500mg effervescent tablets (Advanz Pharma) | | 15 | 0.1 | 1.5 | PCGP |
| codeine/paracetamol | Codipar 15mg/500mg tablets (Advanz Pharma) | | 15 | 0.1 | 1.5 | PCGP |
| codeine/paracetamol | Emcozin 30mg/500mg tablets (M & A Pharmachem Ltd) | | 30 | 0.1 | 3 | PCGP |
| codeine/paracetamol | Kapake 15mg/500mg tablets (Galen Ltd) | | 15 | 0.1 | 1.5 | PCGP |
| codeine/paracetamol | Kapake 30mg/500mg capsules (Galen Ltd) | | 30 | 0.1 | 3 | PCGP |
| codeine/paracetamol | Kapake 30mg/500mg tablets (Galen Ltd) | | 30 | 0.1 | 3 | PCGP |
| codeine/paracetamol | Migraleve Yellow tablets (McNeil Products Ltd) | | 8 | 0.1 | 0.8 | PCGP |
| codeine/paracetamol | Paracodol 8mg/500mg capsules (Bayer Plc) | | 8 | 0.1 | 0.8 | PCGP |
| codeine/paracetamol | Paracodol 8mg/500mg effervescent tablets (Bayer Plc) | | 8 | 0.1 | 0.8 | PCGP |
| codeine/paracetamol | Solpadeine Max 12.8mg/500mg tablets (Omega Pharma Ltd) | | 12.8 | 0.1 | 1.28 | PCGP |
| codeine/paracetamol | Solpadol 30mg/500mg caplets (Sanofi) | | 30 | 0.1 | 3 | PCGP |
| codeine/paracetamol | Solpadol 30mg/500mg capsules (Sanofi) | | 30 | 0.1 | 3 | PCGP |
| codeine/paracetamol | Solpadol 30mg/500mg effervescent tablets (Sanofi) | | 30 | 0.1 | 3 | PCGP |
| codeine/paracetamol | Tylex 30mg/500mg capsules (UCB Pharma Ltd) | | 30 | 0.1 | 3 | PCGP |
| codeine/paracetamol | Tylex 30mg/500mg effervescent tablets (UCB Pharma Ltd) | | 30 | 0.1 | 3 | PCGP |
| codeine/paracetamol | Zapain 30mg/500mg capsules (Advanz Pharma) | | 30 | 0.1 | 3 | PCGP |
| codeine/paracetamol | Zapain 30mg/500mg tablets (Advanz Pharma) | | 30 | 0.1 | 3 | PCGP |
| cyclizine/dipipanone | diconal tablets (amdipharm plc) | | 10 | 0.5 | 5 | OPM |
| cyclizine/dipipanone | dipipanone 10mg / cyclizine 30mg tablets | | 10 | 0.5 | 5 | OPM |
| cyclizine/morphine | cyclimorph 10 solution for injection 1ml ampoules (advanz pharma) | | 10 | 2 | 20 | PCGP |
| cyclizine/morphine | cyclimorph 15 solution for injection 1ml ampoules (advanz pharma) | | 15 | 2 | 30 | PCGP |
| diamorphine | diamorphine hydrochloride injection 10 mg/ml | | 10 | 3 | 30 | PCGP |
| diamorphine | diamorphine hydrochloride injection 5 mg | | 5 | 3 | 15 | PCGP |
| diamorphine | diamorphine hydrochloride injection 500 mg/2 ml | | 250 | 3 | 750 | PCGP |
| diamorphine | diamorphine hydrochloride linctus 3mg/5 ml | | 0.6 | 1 | 0.6 | OPM |
| diamorphine | diamorphine hydrochloride tablets 10 mg | | 10 | 1 | 10 | OPM |
| morphine | morphine and atropine injection 1 ml ampoule | |  | 2 | 0 | PCGP |
| morphine | morphine hydrochloride mixture 916 micrograms | | 0.916 | 1 | 0.916 | PCGP |
| morphine | morphine injection 64mg/ml | | 64 | 2 | 128 | PCGP |
| morphine | morphine oral solution 8.4 mg/ml | | 8.4 | 1 | 8.4 | PCGP |
| morphine | morphine sulfate concentrated oral solution 20 mg/ml | | 20 | 1 | 20 | PCGP |
| morphine | morphine sulfate epidural injection 2 mg/10 ml | | 0.2 | 2 | 0.4 | PCGP |
| morphine | morphine sulfate injection 1 mg/ml, 50 ml syringe | | 50 | 2 | 100 | PCGP |
| morphine | morphine sulfate injection 10 mg/0.7 ml auto-injector | | 7 | 2 | 14 | PCGP |
| morphine | morphine sulfate injection 10 mg/ml | | 10 | 2 | 20 | PCGP |
| morphine | morphine sulfate injection 15 mg/ml | | 15 | 2 | 30 | PCGP |
| morphine | morphine sulfate injection 2 mg/ml, 50 ml syringe | | 100 | 2 | 200 | PCGP |
| morphine | morphine sulfate injection 30 mg/ml | | 30 | 2 | 60 | PCGP |
| morphine | morphine sulfate sachets 20 mg | | 20 | 1 | 20 | PCGP |
| morphine | morphine sulfate sachets 30 mg | | 30 | 1 | 30 | PCGP |
| cyclizine/morphine | morphine tartrate 10mg/1ml / cyclizine tartrate 50mg/1ml solution for injection ampoules | | 10 | 2 | 20 | PCGP |
| cyclizine/morphine | morphine tartrate 15mg/1ml / cyclizine tartrate 50mg/1ml solution for injection ampoules | | 15 | 2 | 30 | PCGP |
| dexketoprofen/tramadol | tramadol 75mg / dexketoprofen 25mg tablets | | 75 | 0.1 | 7.5 | PCGP |
| diamorphine | diamorphine 100mg powder for solution for injection ampoules | | 100 | 3 | 300 | PCGP |
| diamorphine | diamorphine 100mg powder for solution for injection vials | | 100 | 3 | 300 | PCGP |
| diamorphine | diamorphine 10mg powder for solution for injection ampoules | | 10 | 3 | 30 | PCGP |
| diamorphine | diamorphine 10mg powder for solution for injection vials | | 10 | 3 | 30 | PCGP |
| diamorphine | diamorphine 10mg tablets | | 10 | 1 | 10 | OPM |
| diamorphine | diamorphine 25mg/5ml oral solution | | 5 | 1 | 5 | OPM |
| diamorphine | diamorphine 30mg powder for solution for injection ampoules | | 30 | 3 | 90 | PCGP |
| diamorphine | diamorphine 30mg powder for solution for injection vials | | 30 | 3 | 90 | PCGP |
| diamorphine | diamorphine 3mg/5ml oral solution | | 0.6 | 1 | 0.6 | OPM |
| diamorphine | diamorphine 500mg powder for solution for injection ampoules | | 500 | 3 | 1500 | PCGP |
| diamorphine | diamorphine 500mg powder for solution for injection vials | | 500 | 3 | 1500 | PCGP |
| diamorphine | diamorphine 5mg powder for solution for injection ampoules | | 5 | 3 | 15 | PCGP |
| diamorphine | diamorphine 5mg powder for solution for injection vials | | 5 | 3 | 15 | PCGP |
| diamorphine | diamorphine hydrochloride powder | | 1 | 3 | 3 | PCGP |
| dihydrocodeine | df 118 elixir 10 mg/5 ml | | 2 | 0.1 | 0.2 | PCGP |
| dihydrocodeine | df 118 forte 40mg tablets (martindale pharmaceuticals ltd) | | 40 | 0.1 | 4 | PCGP |
| dihydrocodeine | df 118 injection 50 mg/ml | | 50 | 0.3 | 5 | NICE |
| dihydrocodeine | df 118 tablets 30 mg | | 30 | 0.1 | 3 | PCGP |
| dihydrocodeine | dhc continus 120mg tablets (napp pharmaceuticals ltd) | | 120 | 0.1 | 12 | PCGP |
| dihydrocodeine | dhc continus 60mg tablets (napp pharmaceuticals ltd) | | 60 | 0.1 | 6 | PCGP |
| dihydrocodeine | dhc continus 90mg tablets (napp pharmaceuticals ltd) | | 90 | 0.1 | 9 | PCGP |
| dihydrocodeine | dihydrocodeine 10mg/5ml oral solution | | 2 | 0.1 | 0.2 | PCGP |
| dihydrocodeine | dihydrocodeine 10mg/5ml oral suspension | | 2 | 0.1 | 0.2 | PCGP |
| dihydrocodeine | dihydrocodeine 120mg modified-release tablets | | 120 | 0.1 | 12 | PCGP |
| dihydrocodeine | dihydrocodeine 30mg tablets | | 30 | 0.1 | 3 | PCGP |
| dihydrocodeine | dihydrocodeine 40mg tablets | | 40 | 0.1 | 4 | PCGP |
| dihydrocodeine | dihydrocodeine 50mg/1ml solution for injection ampoules | | 50 | 0.3 | 5 | NICE |
| dihydrocodeine | dihydrocodeine 60mg modified-release tablets | | 60 | 0.1 | 6 | PCGP |
| dihydrocodeine | dihydrocodeine 90mg modified-release tablets | | 90 | 0.1 | 9 | PCGP |
| dihydrocodeine/paracetamol | Co-dydramol 10mg/500mg tablets | | 10 | 0.1 | 1 | PCGP |
| dihydrocodeine/paracetamol | Co-dydramol 10mg/500mg/5ml oral solution | | 2 | 0.1 | 0.2 | PCGP |
| dihydrocodeine/paracetamol | Co-dydramol 10mg/500mg/5ml oral suspension | | 2 | 0.1 | 0.2 | PCGP |
| dihydrocodeine/paracetamol | Co-dydramol 20mg/500mg tablets | | 20 | 0.1 | 2 | PCGP |
| dihydrocodeine/paracetamol | Co-dydramol 30mg/500mg tablets | | 30 | 0.1 | 3 | PCGP |
| dihydrocodeine/paracetamol | Dypracet 20mg/500mg tablets (Auden McKenzie (Pharma Division) Ltd) | | 20 | 0.1 | 2 | PCGP |
| dihydrocodeine/paracetamol | Paramol tablets (SSL International Plc) | | 7.46 | 0.1 | 0.746 | PCGP |
| dihydrocodeine/paracetamol | Remedeine Forte tablets (Crescent Pharma Ltd) | | 30 | 0.1 | 3 | PCGP |
| dihydrocodeine/paracetamol | Remedeine tablets (Crescent Pharma Ltd) | | 20 | 0.1 | 2 | PCGP |
| fentanyl | durogesic 100micrograms transdermal patches (janssen-cilag ltd) | | 2.4 | 150 | 360 | PCGP |
| fentanyl | durogesic 25micrograms transdermal patches (janssen-cilag ltd) | | 0.6 | 150 | 90 | PCGP |
| fentanyl | durogesic 50micrograms transdermal patches (janssen-cilag ltd) | | 1.2 | 150 | 180 | PCGP |
| fentanyl | durogesic 75micrograms transdermal patches (janssen-cilag ltd) | | 1.8 | 150 | 270 | PCGP |
| fentanyl | durogesic dtrans 100micrograms/hour transdermal patches (janssen-cilag ltd) | | 2.4 | 150 | 360 | PCGP |
| fentanyl | durogesic dtrans 12micrograms/hour transdermal patches (janssen-cilag ltd) | | 0.288 | 150 | 43.2 | PCGP |
| fentanyl | durogesic dtrans 25micrograms/hour transdermal patches (janssen-cilag ltd) | | 0.6 | 150 | 90 | PCGP |
| fentanyl | durogesic dtrans 50micrograms/hour transdermal patches (janssen-cilag ltd) | | 1.2 | 150 | 180 | PCGP |
| fentanyl | durogesic dtrans 75micrograms/hour transdermal patches (janssen-cilag ltd) | | 1.8 | 150 | 270 | PCGP |
| fentanyl | fencino 100micrograms/hour transdermal patches (ethypharm uk ltd) | | 2.4 | 150 | 360 | PCGP |
| fentanyl | fencino 12micrograms/hour transdermal patches (ethypharm uk ltd) | | 0.288 | 150 | 43.2 | PCGP |
| fentanyl | fencino 25micrograms/hour transdermal patches (ethypharm uk ltd) | | 0.6 | 150 | 90 | PCGP |
| fentanyl | fencino 50micrograms/hour transdermal patches (ethypharm uk ltd) | | 1.2 | 150 | 180 | PCGP |
| fentanyl | fencino 75micrograms/hour transdermal patches (ethypharm uk ltd) | | 1.8 | 150 | 270 | PCGP |
| fentanyl | fentalis reservoir 100micrograms/hour transdermal patches (sandoz ltd) | | 2.4 | 150 | 360 | PCGP |
| fentanyl | fentalis reservoir 25micrograms/hour transdermal patches (sandoz ltd) | | 0.6 | 150 | 90 | PCGP |
| fentanyl | fentalis reservoir 50micrograms/hour transdermal patches (sandoz ltd) | | 1.2 | 150 | 180 | PCGP |
| fentanyl | fentalis reservoir 75micrograms/hour transdermal patches (sandoz ltd) | | 1.8 | 150 | 270 | PCGP |
| fentanyl | fentanyl 100micrograms/hour transdermal patches | | 2.4 | 150 | 360 | PCGP |
| fentanyl | fentanyl 12micrograms/hour transdermal patches | | 0.288 | 150 | 43.2 | PCGP |
| fentanyl | fentanyl 25micrograms/hour transdermal patches | | 0.6 | 150 | 90 | PCGP |
| fentanyl | fentanyl 37.5microgram/hour transdermal patches | | 0.9 | 150 | 135 | PCGP |
| fentanyl | fentanyl 40micrograms/dose transdermal system | | 0.04 | 150 | 6 | PCGP |
| fentanyl | fentanyl 50micrograms/hour transdermal patches | | 1.2 | 150 | 180 | PCGP |
| fentanyl | fentanyl 75micrograms/hour transdermal patches | | 1.8 | 150 | 270 | PCGP |
| fentanyl | fentanyl citrate injection 2.5 mg/ml | | 2.5 | 150 | 375 | PCGP |
| fentanyl | fentanyl citrate injection 50 micrograms/ml | | 0.05 | 150 | 7.5 | PCGP |
| fentanyl | fentanyl solution for injection 100 micrograms/2 ml ampoule | | 0.05 | 150 | 7.5 | PCGP |
| fentanyl | ionsys 40micrograms/dose transdermal system (the medicines company uk ltd) | | 0.04 | 150 | 6 | PCGP |
| fentanyl | matrifen 100micrograms/hour transdermal patches (teva uk ltd) | | 2.4 | 150 | 360 | PCGP |
| fentanyl | matrifen 12micrograms/hour transdermal patches (teva uk ltd) | | 0.288 | 150 | 43.2 | PCGP |
| fentanyl | matrifen 25micrograms/hour transdermal patches (teva uk ltd) | | 0.6 | 150 | 90 | PCGP |
| fentanyl | matrifen 50micrograms/hour transdermal patches (teva uk ltd) | | 1.2 | 150 | 180 | PCGP |
| fentanyl | matrifen 75micrograms/hour transdermal patches (teva uk ltd) | | 1.8 | 150 | 270 | PCGP |
| fentanyl | mezolar matrix 100micrograms/hour transdermal patches (sandoz ltd) | | 2.4 | 150 | 360 | PCGP |
| fentanyl | mezolar matrix 12micrograms/hour transdermal patches (sandoz ltd) | | 0.288 | 150 | 43.2 | PCGP |
| fentanyl | mezolar matrix 25micrograms/hour transdermal patches (sandoz ltd) | | 0.6 | 150 | 90 | PCGP |
| fentanyl | mezolar matrix 37.5microgram/hour transdermal patches (sandoz ltd) | | 0.9 | 150 | 135 | PCGP |
| fentanyl | mezolar matrix 50micrograms/hour transdermal patches (sandoz ltd) | | 1.2 | 150 | 180 | PCGP |
| fentanyl | mezolar matrix 75micrograms/hour transdermal patches (sandoz ltd) | | 1.8 | 150 | 270 | PCGP |
| fentanyl | mylafent 100micrograms/hour transdermal patches (mylan) | | 2.4 | 150 | 360 | PCGP |
| fentanyl | mylafent 12micrograms/hour transdermal patches (mylan) | | 0.288 | 150 | 43.2 | PCGP |
| fentanyl | mylafent 25micrograms/hour transdermal patches (mylan) | | 0.6 | 150 | 90 | PCGP |
| fentanyl | mylafent 50micrograms/hour transdermal patches (mylan) | | 1.2 | 150 | 180 | PCGP |
| fentanyl | mylafent 75micrograms/hour transdermal patches (mylan) | | 1.8 | 150 | 270 | PCGP |
| fentanyl | opiodur 100micrograms/hour transdermal patches (zentiva) | | 2.4 | 150 | 360 | PCGP |
| fentanyl | opiodur 12micrograms/hour transdermal patches (zentiva) | | 0.288 | 150 | 43.2 | PCGP |
| fentanyl | opiodur 25micrograms/hour transdermal patches (zentiva) | | 0.6 | 150 | 90 | PCGP |
| fentanyl | opiodur 50micrograms/hour transdermal patches (zentiva) | | 1.2 | 150 | 180 | PCGP |
| fentanyl | opiodur 75micrograms/hour transdermal patches (zentiva) | | 1.8 | 150 | 270 | PCGP |
| fentanyl | osmach 100micrograms/hour transdermal patches (ratiopharm uk ltd) | | 2.4 | 150 | 360 | PCGP |
| fentanyl | osmach 25micrograms/hour transdermal patches (teva uk ltd) | | 0.6 | 150 | 90 | PCGP |
| fentanyl | osmach 50micrograms/hour transdermal patches (teva uk ltd) | | 1.2 | 150 | 180 | PCGP |
| fentanyl | osmach 75micrograms/hour transdermal patches (teva uk ltd) | | 1.8 | 150 | 270 | PCGP |
| fentanyl | osmanil 100micrograms/hour transdermal patches (zentiva) | | 2.4 | 150 | 360 | PCGP |
| fentanyl | osmanil 12micrograms/hour transdermal patches (zentiva) | | 0.288 | 150 | 43.2 | PCGP |
| fentanyl | osmanil 25micrograms/hour transdermal patches (zentiva) | | 0.6 | 150 | 90 | PCGP |
| fentanyl | osmanil 50micrograms/hour transdermal patches (zentiva) | | 1.2 | 150 | 180 | PCGP |
| fentanyl | osmanil 75micrograms/hour transdermal patches (zentiva) | | 1.8 | 150 | 270 | PCGP |
| fentanyl | tilofyl 100micrograms/hour transdermal patches (tillomed laboratories ltd) | | 2.4 | 150 | 360 | PCGP |
| fentanyl | tilofyl 25micrograms/hour transdermal patches (tillomed laboratories ltd) | | 0.6 | 150 | 90 | PCGP |
| fentanyl | tilofyl 50micrograms/hour transdermal patches (tillomed laboratories ltd) | | 1.2 | 150 | 180 | PCGP |
| fentanyl | tilofyl 75micrograms/hour transdermal patches (tillomed laboratories ltd) | | 1.8 | 150 | 270 | PCGP |
| fentanyl | victanyl 100micrograms/hour transdermal patches (accord healthcare ltd) | | 2.4 | 150 | 360 | PCGP |
| fentanyl | victanyl 12micrograms/hour transdermal patches (accord healthcare ltd) | | 0.288 | 150 | 43.2 | PCGP |
| fentanyl | victanyl 25micrograms/hour transdermal patches (accord healthcare ltd) | | 0.6 | 150 | 90 | PCGP |
| fentanyl | victanyl 50micrograms/hour transdermal patches (accord healthcare ltd) | | 1.2 | 150 | 180 | PCGP |
| fentanyl | victanyl 75micrograms/hour transdermal patches (accord healthcare ltd) | | 1.8 | 150 | 270 | PCGP |
| fentanyl | yemex 100micrograms/hour transdermal patches (sandoz ltd) | | 2.4 | 150 | 360 | PCGP |
| fentanyl | yemex 12micrograms/hour transdermal patches (sandoz ltd) | | 0.288 | 150 | 43.2 | PCGP |
| fentanyl | yemex 25micrograms/hour transdermal patches (sandoz ltd) | | 0.6 | 150 | 90 | PCGP |
| fentanyl | yemex 50micrograms/hour transdermal patches (sandoz ltd) | | 1.2 | 150 | 180 | PCGP |
| fentanyl | yemex 75micrograms/hour transdermal patches (sandoz ltd) | | 1.8 | 150 | 270 | PCGP |
| fentanyl | fentanyl 100micrograms/2ml solution for injection ampoules | | 0.05 | 150 | 7.5 | PCGP |
| fentanyl | fentanyl 2.5mg/50ml solution for infusion vials | | 0.05 | 150 | 7.5 | PCGP |
| fentanyl | fentanyl 500micrograms/10ml solution for injection ampoules | | 0.05 | 150 | 7.5 | PCGP |
| fentanyl | sublimaze 100micrograms/2ml solution for injection ampoules (janssen-cilag ltd) | | 0.05 | 150 | 7.5 | PCGP |
| fentanyl | sublimaze 500micrograms/10ml solution for injection ampoules (piramal critical care ltd) | | 0.05 | 150 | 7.5 | PCGP |
| fentanyl | sublimaze injection 50 micrograms/ml | | 0.05 | 150 | 7.5 | PCGP |
| hydromorphone | hydromorphone 1.3mg capsules | | 1.3 | 7.5 | 9.75 | PCGP |
| hydromorphone | hydromorphone 10mg/1ml solution for injection ampoules | | 10 | 15 | 150 | PCGP |
| hydromorphone | hydromorphone 16mg modified-release capsules | | 16 | 7.5 | 120 | PCGP |
| hydromorphone | hydromorphone 2.6mg capsules | | 2.6 | 7.5 | 19.5 | PCGP |
| hydromorphone | hydromorphone 20mg/1ml solution for injection ampoules | | 20 | 15 | 300 | PCGP |
| hydromorphone | hydromorphone 24mg modified-release capsules | | 24 | 7.5 | 180 | PCGP |
| hydromorphone | hydromorphone 2mg modified-release capsules | | 2 | 7.5 | 15 | PCGP |
| hydromorphone | hydromorphone 2mg/1ml solution for injection ampoules | | 2 | 15 | 30 | PCGP |
| hydromorphone | hydromorphone 4mg modified-release capsules | | 4 | 7.5 | 30 | PCGP |
| hydromorphone | hydromorphone 50mg/1ml solution for injection ampoules | | 50 | 15 | 750 | PCGP |
| hydromorphone | hydromorphone 8mg modified-release capsules | | 8 | 7.5 | 60 | PCGP |
| hydromorphone | palladone 1.3mg capsules (napp pharmaceuticals ltd) | | 1.3 | 7.5 | 9.75 | PCGP |
| hydromorphone | palladone 10mg/1ml solution for injection ampoules (napp pharmaceuticals ltd) | | 10 | 15 | 150 | PCGP |
| hydromorphone | palladone 2.6mg capsules (napp pharmaceuticals ltd) | | 2.6 | 7.5 | 19.5 | PCGP |
| hydromorphone | palladone 20mg/1ml solution for injection ampoules (napp pharmaceuticals ltd) | | 20 | 15 | 300 | PCGP |
| hydromorphone | palladone 2mg/1ml solution for injection ampoules (napp pharmaceuticals ltd) | | 2 | 15 | 30 | PCGP |
| hydromorphone | palladone 50mg/1ml solution for injection ampoules (napp pharmaceuticals ltd) | | 50 | 15 | 750 | PCGP |
| hydromorphone | palladone sr 16mg capsules (napp pharmaceuticals ltd) | | 16 | 7.5 | 120 | PCGP |
| hydromorphone | palladone sr 24mg capsules (napp pharmaceuticals ltd) | | 24 | 7.5 | 180 | PCGP |
| hydromorphone | palladone sr 2mg capsules (napp pharmaceuticals ltd) | | 2 | 7.5 | 15 | PCGP |
| hydromorphone | palladone sr 4mg capsules (napp pharmaceuticals ltd) | | 4 | 7.5 | 30 | PCGP |
| hydromorphone | palladone sr 8mg capsules (napp pharmaceuticals ltd) | | 8 | 7.5 | 60 | PCGP |
| meptazinol | meptazinol 200mg tablets | | 200 | 0.13 | 26 | OPM |
| meptazinol | meptid 200mg tablets (almirall ltd) | | 200 | 0.13 | 26 | OPM |
| methadone | eptadone 1mg/ml oral solution (dee pharmaceuticals ltd) | | 1 | 4 | 4 | OMCF |
| methadone | eptadone 5mg/ml oral solution (dee pharmaceuticals ltd) | | 5 | 4 | 20 | OMCF |
| methadone | methadone 100mg capsules | | 100 | 4 | 400 | OMCF |
| methadone | methadone 10mg/1ml solution for injection ampoules | | 10 | 5 | 50 | UCSF |
| methadone | methadone 10mg/ml oral solution sugar free | | 10 | 4 | 40 | OMCF |
| methadone | methadone 1mg/ml oral solution | | 1 | 4 | 4 | OMCF |
| methadone | methadone 1mg/ml oral solution sugar free | | 1 | 4 | 4 | OMCF |
| methadone | methadone 20mg/2ml solution for injection ampoules | | 10 | 5 | 50 | UCSF |
| methadone | methadone 20mg/ml oral solution sugar free | | 20 | 4 | 80 | OMCF |
| methadone | methadone 2mg/5ml linctus | | 0.4 | 4 | 1.6 | OMCF |
| methadone | methadone 30mg capsules | | 30 | 4 | 120 | OMCF |
| methadone | methadone 35mg/3.5ml solution for injection ampoules | | 10 | 5 | 50 | UCSF |
| methadone | methadone 50mg/1ml solution for injection ampoules | | 50 | 5 | 250 | UCSF |
| methadone | methadone 50mg/2ml solution for injection ampoules | | 25 | 5 | 125 | UCSF |
| methadone | methadone 50mg/5ml solution for injection ampoules | | 10 | 5 | 50 | UCSF |
| methadone | methadone 5mg capsules | | 5 | 4 | 20 | OMCF |
| methadone | methadone 5mg tablets | | 5 | 4 | 20 | OMCF |
| methadone | methadone hydrochloride diluent oral solution sugar free | | 1 | 4 | 4 | OMCF |
| methadone | methadone hydrochloride injection 10mg/ml | | 10 | 5 | 50 | UCSF |
| methadone | methadone hydrochloride oral solution, sugar free, tartrazine free 1 mg/1 ml | | 1 | 4 | 4 | OMCF |
| methadone | methadone hydrochloride powder | | 1 | 4 | 4 | OMCF |
| methadone | methadose 10mg/ml oral solution concentrate (rosemont pharmaceuticals ltd) | | 10 | 4 | 40 | OMCF |
| methadone | methadose 20mg/ml oral solution concentrate (rosemont pharmaceuticals ltd) | | 20 | 4 | 80 | OMCF |
| methadone | methadose diluent oral solution sugar free (rosemont pharmaceuticals ltd) | | 1 | 4 | 4 | OMCF |
| methadone | metharose 1mg/ml oral solution sugar free (rosemont pharmaceuticals ltd) | | 1 | 4 | 4 | OMCF |
| methadone | physeptone 10mg/1ml solution for injection ampoules (martindale pharmaceuticals ltd) | | 10 | 5 | 50 | UCSF |
| methadone | physeptone 1mg/ml mixture (martindale pharmaceuticals ltd) | | 1 | 4 | 4 | OMCF |
| methadone | physeptone 1mg/ml oral solution sugar free (martindale pharmaceuticals ltd) | | 1 | 4 | 4 | OMCF |
| methadone | physeptone 20mg/2ml solution for injection ampoules (martindale pharmaceuticals ltd) | | 10 | 5 | 50 | UCSF |
| methadone | physeptone 35mg/3.5ml solution for injection ampoules (martindale pharmaceuticals ltd) | | 10 | 5 | 50 | UCSF |
| methadone | physeptone 50mg/1ml solution for injection ampoules (martindale pharmaceuticals ltd) | | 50 | 5 | 250 | UCSF |
| methadone | physeptone 50mg/2ml solution for injection ampoules (martindale pharmaceuticals ltd) | | 25 | 5 | 125 | UCSF |
| methadone | physeptone 50mg/5ml solution for injection ampoules (martindale pharmaceuticals ltd) | | 10 | 5 | 50 | UCSF |
| methadone | physeptone 5mg tablets (martindale pharmaceuticals ltd) | | 5 | 4 | 20 | OMCF |
| methadone | physeptone injection 10mg/ml | | 10 | 5 | 50 | UCSF |
| methadone | physeptone linctus 2 mg/5 ml | | 0.4 | 4 | 1.6 | OMCF |
| methadone | synastone 10mg/1ml solution for injection ampoules (auden mckenzie (pharma division) ltd) | | 10 | 5 | 50 | UCSF |
| methadone | synastone 50mg/1ml solution for injection ampoules (auden mckenzie (pharma division) ltd) | | 50 | 5 | 250 | UCSF |
| methadone | synastone 50mg/2ml solution for injection ampoules (auden mckenzie (pharma division) ltd) | | 25 | 5 | 125 | UCSF |
| methadone | synastone 50mg/5ml solution for injection ampoules (auden mckenzie (pharma division) ltd) | | 10 | 5 | 50 | UCSF |
| methadone | synastone injection 10 mg/ml, 2 ml ampoule | | 20 | 5 | 100 | UCSF |
| methadone | synastone injection 10 mg/ml, 3.5 ml ampoule | | 35 | 5 | 175 | UCSF |
| morphine | morphine (opium tincture) 10mg/ml oral drops sugar free | | 10 | 1 | 10 | PCGP |
| morphine | morphine hydrochloride 10mg/5ml oral solution | | 2 | 1 | 2 | PCGP |
| morphine | morphine hydrochloride powder | | 1 | 1 | 1 | PCGP |
| morphine | depodur 10mg/1ml suspension for injection vials (flynn pharma ltd) | | 10 | 30 | 300 | UCSF |
| morphine | filnarine sr 100mg tablets (teva uk ltd) | | 100 | 1 | 100 | PCGP |
| morphine | filnarine sr 10mg tablets (teva uk ltd) | | 10 | 1 | 10 | PCGP |
| morphine | filnarine sr 200mg tablets (teva uk ltd) | | 200 | 1 | 200 | PCGP |
| morphine | filnarine sr 30mg tablets (teva uk ltd) | | 30 | 1 | 30 | PCGP |
| morphine | filnarine sr 60mg tablets (teva uk ltd) | | 60 | 1 | 60 | PCGP |
| morphine | morcap sr 100mg capsules (hospira uk ltd) | | 100 | 1 | 100 | PCGP |
| morphine | morcap sr 20mg capsules (hospira uk ltd) | | 20 | 1 | 20 | PCGP |
| morphine | morcap sr 50mg capsules (hospira uk ltd) | | 50 | 1 | 50 | PCGP |
| morphine | morphgesic sr 100mg tablets (advanz pharma) | | 100 | 1 | 100 | PCGP |
| morphine | morphgesic sr 10mg tablets (advanz pharma) | | 10 | 1 | 10 | PCGP |
| morphine | morphgesic sr 30mg tablets (advanz pharma) | | 30 | 1 | 30 | PCGP |
| morphine | morphgesic sr 60mg tablets (advanz pharma) | | 60 | 1 | 60 | PCGP |
| morphine | morphine 100mg modified-release capsules | | 100 | 1 | 100 | PCGP |
| morphine | morphine 100mg modified-release granules sachets sugar free | | 100 | 1 | 100 | PCGP |
| morphine | morphine 100mg modified-release tablets | | 100 | 1 | 100 | PCGP |
| morphine | morphine 10mg modified-release capsules | | 10 | 1 | 10 | PCGP |
| morphine | morphine 10mg modified-release tablets | | 10 | 1 | 10 | PCGP |
| morphine | morphine 10mg tablets | | 10 | 1 | 10 | PCGP |
| morphine | morphine 120mg modified-release capsules | | 120 | 1 | 120 | PCGP |
| morphine | morphine 150mg modified-release capsules | | 150 | 1 | 150 | PCGP |
| morphine | morphine 15mg modified-release tablets | | 15 | 1 | 15 | PCGP |
| morphine | morphine 200mg modified-release capsules | | 200 | 1 | 200 | PCGP |
| morphine | morphine 200mg modified-release granules sachets sugar free | | 200 | 1 | 200 | PCGP |
| morphine | morphine 200mg modified-release tablets | | 200 | 1 | 200 | PCGP |
| morphine | morphine 20mg modified-release capsules | | 20 | 1 | 20 | PCGP |
| morphine | morphine 20mg modified-release granules sachets sugar free | | 20 | 1 | 20 | PCGP |
| morphine | morphine 20mg tablets | | 20 | 1 | 20 | PCGP |
| morphine | morphine 30mg modified-release capsules | | 30 | 1 | 30 | PCGP |
| morphine | morphine 30mg modified-release granules sachets sugar free | | 30 | 1 | 30 | PCGP |
| morphine | morphine 30mg modified-release tablets | | 30 | 1 | 30 | PCGP |
| morphine | morphine 50mg modified-release capsules | | 50 | 1 | 50 | PCGP |
| morphine | morphine 50mg tablets | | 50 | 1 | 50 | PCGP |
| morphine | morphine 5mg modified-release tablets | | 5 | 1 | 5 | PCGP |
| morphine | morphine 60mg modified-release capsules | | 60 | 1 | 60 | PCGP |
| morphine | morphine 60mg modified-release granules sachets sugar free | | 60 | 1 | 60 | PCGP |
| morphine | morphine 60mg modified-release tablets | | 60 | 1 | 60 | PCGP |
| morphine | morphine 90mg modified-release capsules | | 90 | 1 | 90 | PCGP |
| morphine | morphine sulfate 100mg/50ml solution for infusion vials | | 2 | 3 | 6 | UCSF |
| morphine | morphine sulfate 100mg/5ml oral solution unit dose vials sugar free | | 20 | 1 | 20 | PCGP |
| morphine | morphine sulfate 10mg/10ml solution for injection ampoules | | 1 | 3 | 3 | UCSF |
| morphine | morphine sulfate 10mg/10ml solution for injection minijet pre-filled syringes (ucb pharma ltd) | | 1 | 2 | 2 | PCGP |
| morphine | morphine sulfate 10mg/10ml solution for injection pre-filled syringes | | 1 | 2 | 2 | PCGP |
| morphine | morphine sulfate 10mg/1ml solution for injection ampoules | | 10 | 2 | 20 | PCGP |
| morphine | morphine sulfate 10mg/1ml suspension for injection vials | | 10 | 30 | 300 | UCSF |
| morphine | morphine sulfate 10mg/5ml oral solution | | 2 | 1 | 2 | PCGP |
| morphine | morphine sulfate 10mg/5ml oral solution unit dose vials sugar free | | 2 | 1 | 2 | PCGP |
| morphine | morphine sulfate 15mg/1ml solution for injection ampoules | | 15 | 2 | 30 | PCGP |
| morphine | morphine sulfate 1mg/1ml solution for injection ampoules | | 1 | 3 | 3 | UCSF |
| morphine | morphine sulfate 20mg/1ml solution for injection ampoules | | 20 | 2 | 40 | PCGP |
| morphine | morphine sulfate 20mg/ml oral solution sugar free | | 20 | 1 | 20 | PCGP |
| morphine | morphine sulfate 30mg/1ml solution for injection ampoules | | 30 | 2 | 60 | PCGP |
| morphine | morphine sulfate 30mg/5ml oral solution unit dose vials sugar free | | 6 | 1 | 6 | PCGP |
| morphine | morphine sulfate 50mg/50ml solution for infusion vials | | 1 | 3 | 3 | UCSF |
| morphine | morphine sulfate 5mg/5ml oral solution | | 1 | 1 | 1 | PCGP |
| morphine | morphine sulfate 5mg/5ml solution for injection ampoules | | 1 | 3 | 3 | UCSF |
| morphine | morphine sulfate 60mg/2ml solution for injection ampoules | | 30 | 2 | 60 | PCGP |
| morphine | morphine sulfate powder | | 1 | 1 | 1 | PCGP |
| morphine | mst continus 100mg tablets (napp pharmaceuticals ltd) | | 100 | 1 | 100 | PCGP |
| morphine | mst continus 10mg tablets (napp pharmaceuticals ltd) | | 10 | 1 | 10 | PCGP |
| morphine | mst continus 15mg tablets (napp pharmaceuticals ltd) | | 15 | 1 | 15 | PCGP |
| morphine | mst continus 200mg tablets (napp pharmaceuticals ltd) | | 200 | 1 | 200 | PCGP |
| morphine | mst continus 30mg tablets (napp pharmaceuticals ltd) | | 30 | 1 | 30 | PCGP |
| morphine | mst continus 5mg tablets (napp pharmaceuticals ltd) | | 5 | 1 | 5 | PCGP |
| morphine | mst continus 60mg tablets (napp pharmaceuticals ltd) | | 60 | 1 | 60 | PCGP |
| morphine | mst continus suspension 100mg granules sachets (napp pharmaceuticals ltd) | | 100 | 1 | 100 | PCGP |
| morphine | mst continus suspension 200mg granules sachets (napp pharmaceuticals ltd) | | 200 | 1 | 200 | PCGP |
| morphine | mst continus suspension 20mg granules sachets (napp pharmaceuticals ltd) | | 20 | 1 | 20 | PCGP |
| morphine | mst continus suspension 30mg granules sachets (napp pharmaceuticals ltd) | | 30 | 1 | 30 | PCGP |
| morphine | mst continus suspension 60mg granules sachets (napp pharmaceuticals ltd) | | 60 | 1 | 60 | PCGP |
| morphine | mxl 120mg capsules (napp pharmaceuticals ltd) | | 120 | 1 | 120 | PCGP |
| morphine | mxl 150mg capsules (napp pharmaceuticals ltd) | | 150 | 1 | 150 | PCGP |
| morphine | mxl 200mg capsules (napp pharmaceuticals ltd) | | 200 | 1 | 200 | PCGP |
| morphine | mxl 30mg capsules (napp pharmaceuticals ltd) | | 30 | 1 | 30 | PCGP |
| morphine | mxl 60mg capsules (napp pharmaceuticals ltd) | | 60 | 1 | 60 | PCGP |
| morphine | mxl 90mg capsules (napp pharmaceuticals ltd) | | 90 | 1 | 90 | PCGP |
| morphine | oramorph 100mg/5ml oral solution unit dose vials (boehringer ingelheim ltd) | | 20 | 1 | 20 | PCGP |
| morphine | oramorph 10mg/5ml oral solution (boehringer ingelheim ltd) | | 2 | 1 | 2 | PCGP |
| morphine | oramorph 10mg/5ml oral solution unit dose vials (boehringer ingelheim ltd) | | 2 | 1 | 2 | PCGP |
| morphine | oramorph 20mg/ml concentrated oral solution (boehringer ingelheim ltd) | | 20 | 1 | 20 | PCGP |
| morphine | oramorph 30mg/5ml oral solution unit dose vials (boehringer ingelheim ltd) | | 6 | 1 | 6 | PCGP |
| morphine | oramorph sr m/r tablets 10 mg | | 10 | 1 | 10 | PCGP |
| morphine | oramorph sr m/r tablets 100 mg | | 100 | 1 | 100 | PCGP |
| morphine | oramorph sr m/r tablets 30 mg | | 30 | 1 | 30 | PCGP |
| morphine | oramorph sr m/r tablets 60 mg | | 60 | 1 | 60 | PCGP |
| morphine | sevredol 10mg tablets (napp pharmaceuticals ltd) | | 10 | 1 | 10 | PCGP |
| morphine | sevredol 10mg/5ml oral solution (napp pharmaceuticals ltd) | | 2 | 1 | 2 | PCGP |
| morphine | sevredol 20mg tablets (napp pharmaceuticals ltd) | | 20 | 1 | 20 | PCGP |
| morphine | sevredol 20mg/ml concentrated oral solution (napp pharmaceuticals ltd) | | 20 | 1 | 20 | PCGP |
| morphine | sevredol 50mg tablets (napp pharmaceuticals ltd) | | 50 | 1 | 50 | PCGP |
| morphine | zomorph 100mg modified-release capsules (ethypharm uk ltd) | | 100 | 1 | 100 | PCGP |
| morphine | zomorph 10mg modified-release capsules (ethypharm uk ltd) | | 10 | 1 | 10 | PCGP |
| morphine | zomorph 200mg modified-release capsules (ethypharm uk ltd) | | 200 | 1 | 200 | PCGP |
| morphine | zomorph 30mg modified-release capsules (ethypharm uk ltd) | | 30 | 1 | 30 | PCGP |
| morphine | zomorph 60mg modified-release capsules (ethypharm uk ltd) | | 60 | 1 | 60 | PCGP |
| naloxone/oxycodone | myloxifin 10mg/5mg modified-release tablets (zentiva) | | 2 | 2 | 4 | PCGP |
| naloxone/oxycodone | myloxifin 20mg/10mg modified-release tablets (zentiva) | | 4 | 2 | 8 | PCGP |
| naloxone/oxycodone | myloxifin 40mg/20mg modified-release tablets (zentiva) | | 2 | 2 | 4 | PCGP |
| naloxone/oxycodone | myloxifin 5mg/2.5mg modified-release tablets (zentiva) | | 2 | 2 | 4 | PCGP |
| naloxone/oxycodone | oxyargin 10mg/5mg modified-release tablets (mylan) | | 2 | 2 | 4 | PCGP |
| naloxone/oxycodone | oxyargin 20mg/10mg modified-release tablets (mylan) | | 4 | 2 | 8 | PCGP |
| naloxone/oxycodone | oxyargin 40mg/20mg modified-release tablets (mylan) | | 2 | 2 | 4 | PCGP |
| naloxone/oxycodone | oxyargin 5mg/2.5mg modified-release tablets (mylan) | | 2 | 2 | 4 | PCGP |
| naloxone/oxycodone | oxycodone 10mg / naloxone 5mg modified-release tablets | | 10 | 2 | 20 | PCGP |
| naloxone/oxycodone | oxycodone 20mg / naloxone 10mg modified-release tablets | | 20 | 2 | 40 | PCGP |
| naloxone/oxycodone | oxycodone 40mg / naloxone 20mg modified-release tablets | | 40 | 2 | 80 | PCGP |
| naloxone/oxycodone | oxycodone 5mg / naloxone 2.5mg modified-release tablets | | 5 | 2 | 10 | PCGP |
| naloxone/oxycodone | targinact 10mg/5mg modified-release tablets (napp pharmaceuticals ltd) | | 10 | 2 | 20 | PCGP |
| naloxone/oxycodone | targinact 20mg/10mg modified-release tablets (napp pharmaceuticals ltd) | | 20 | 2 | 40 | PCGP |
| naloxone/oxycodone | targinact 40mg/20mg modified-release tablets (napp pharmaceuticals ltd) | | 40 | 2 | 80 | PCGP |
| naloxone/oxycodone | targinact 5mg/2.5mg modified-release tablets (napp pharmaceuticals ltd) | | 5 | 2 | 10 | PCGP |
| oxycodone | abtard 10mg modified-release tablets (ethypharm uk ltd) | | 10 | 2 | 20 | PCGP |
| oxycodone | abtard 15mg modified-release tablets (ethypharm uk ltd) | | 15 | 2 | 30 | PCGP |
| oxycodone | abtard 20mg modified-release tablets (ethypharm uk ltd) | | 20 | 2 | 40 | PCGP |
| oxycodone | abtard 30mg modified-release tablets (ethypharm uk ltd) | | 30 | 2 | 60 | PCGP |
| oxycodone | abtard 40mg modified-release tablets (ethypharm uk ltd) | | 40 | 2 | 80 | PCGP |
| oxycodone | abtard 5mg modified-release tablets (ethypharm uk ltd) | | 5 | 2 | 10 | PCGP |
| oxycodone | abtard 60mg modified-release tablets (ethypharm uk ltd) | | 60 | 2 | 120 | PCGP |
| oxycodone | abtard 80mg modified-release tablets (ethypharm uk ltd) | | 80 | 2 | 160 | PCGP |
| oxycodone | carexil 10mg modified-release tablets (sandoz ltd) | | 10 | 2 | 20 | PCGP |
| oxycodone | carexil 20mg modified-release tablets (sandoz ltd) | | 20 | 2 | 40 | PCGP |
| oxycodone | carexil 40mg modified-release tablets (sandoz ltd) | | 40 | 2 | 80 | PCGP |
| oxycodone | carexil 5mg modified-release tablets (sandoz ltd) | | 5 | 2 | 10 | PCGP |
| oxycodone | carexil 80mg modified-release tablets (sandoz ltd) | | 80 | 2 | 160 | PCGP |
| oxycodone | dolocodon pr 10mg tablets (zentiva) | | 10 | 2 | 20 | PCGP |
| oxycodone | dolocodon pr 20mg tablets (zentiva) | | 20 | 2 | 40 | PCGP |
| oxycodone | dolocodon pr 40mg tablets (zentiva) | | 40 | 2 | 80 | PCGP |
| oxycodone | dolocodon pr 5mg tablets (zentiva) | | 5 | 2 | 10 | PCGP |
| oxycodone | ixyldone 10mg modified-release tablets (morningside healthcare ltd) | | 10 | 2 | 20 | PCGP |
| oxycodone | ixyldone 15mg modified-release tablets (morningside healthcare ltd) | | 15 | 2 | 30 | PCGP |
| oxycodone | ixyldone 20mg modified-release tablets (morningside healthcare ltd) | | 20 | 2 | 40 | PCGP |
| oxycodone | ixyldone 30mg modified-release tablets (morningside healthcare ltd) | | 30 | 2 | 60 | PCGP |
| oxycodone | ixyldone 40mg modified-release tablets (morningside healthcare ltd) | | 40 | 2 | 80 | PCGP |
| oxycodone | ixyldone 5mg modified-release tablets (morningside healthcare ltd) | | 5 | 2 | 10 | PCGP |
| oxycodone | ixyldone 60mg modified-release tablets (morningside healthcare ltd) | | 60 | 2 | 120 | PCGP |
| oxycodone | ixyldone 80mg modified-release tablets (morningside healthcare ltd) | | 80 | 2 | 160 | PCGP |
| oxycodone | leveraxo 10mg modified-release tablets (mylan) | | 10 | 2 | 20 | PCGP |
| oxycodone | leveraxo 20mg modified-release tablets (mylan) | | 20 | 2 | 40 | PCGP |
| oxycodone | leveraxo 30mg modified-release tablets (mylan) | | 30 | 2 | 60 | PCGP |
| oxycodone | leveraxo 40mg modified-release tablets (mylan) | | 40 | 2 | 80 | PCGP |
| oxycodone | leveraxo 5mg modified-release tablets (mylan) | | 5 | 2 | 10 | PCGP |
| oxycodone | leveraxo 60mg modified-release tablets (mylan) | | 60 | 2 | 120 | PCGP |
| oxycodone | leveraxo 80mg modified-release tablets (mylan) | | 80 | 2 | 160 | PCGP |
| oxycodone | longtec 10mg modified-release tablets (qdem pharmaceuticals ltd) | | 10 | 2 | 20 | PCGP |
| oxycodone | longtec 120mg modified-release tablets (qdem pharmaceuticals ltd) | | 120 | 2 | 240 | PCGP |
| oxycodone | longtec 15mg modified-release tablets (qdem pharmaceuticals ltd) | | 15 | 2 | 30 | PCGP |
| oxycodone | longtec 20mg modified-release tablets (qdem pharmaceuticals ltd) | | 20 | 2 | 40 | PCGP |
| oxycodone | longtec 30mg modified-release tablets (qdem pharmaceuticals ltd) | | 30 | 2 | 60 | PCGP |
| oxycodone | longtec 40mg modified-release tablets (qdem pharmaceuticals ltd) | | 40 | 2 | 80 | PCGP |
| oxycodone | longtec 5mg modified-release tablets (qdem pharmaceuticals ltd) | | 5 | 2 | 10 | PCGP |
| oxycodone | longtec 60mg modified-release tablets (qdem pharmaceuticals ltd) | | 60 | 2 | 120 | PCGP |
| oxycodone | longtec 80mg modified-release tablets (qdem pharmaceuticals ltd) | | 80 | 2 | 160 | PCGP |
| oxycodone | lynlor 10mg capsules (accord healthcare ltd) | | 10 | 2 | 20 | PCGP |
| oxycodone | lynlor 20mg capsules (accord healthcare ltd) | | 20 | 2 | 40 | PCGP |
| oxycodone | lynlor 5mg capsules (accord healthcare ltd) | | 5 | 2 | 10 | PCGP |
| oxycodone | onexila xl 10mg tablets (aspire pharma ltd) | | 10 | 2 | 20 | PCGP |
| oxycodone | onexila xl 20mg tablets (aspire pharma ltd) | | 20 | 2 | 40 | PCGP |
| oxycodone | onexila xl 40mg tablets (aspire pharma ltd) | | 40 | 2 | 80 | PCGP |
| oxycodone | onexila xl 80mg tablets (aspire pharma ltd) | | 80 | 2 | 160 | PCGP |
| oxycodone | oxeltra 10mg modified-release tablets (wockhardt uk ltd) | | 10 | 2 | 20 | PCGP |
| oxycodone | oxeltra 15mg modified-release tablets (wockhardt uk ltd) | | 15 | 2 | 30 | PCGP |
| oxycodone | oxeltra 20mg modified-release tablets (wockhardt uk ltd) | | 20 | 2 | 40 | PCGP |
| oxycodone | oxeltra 30mg modified-release tablets (wockhardt uk ltd) | | 30 | 2 | 60 | PCGP |
| oxycodone | oxeltra 40mg modified-release tablets (wockhardt uk ltd) | | 40 | 2 | 80 | PCGP |
| oxycodone | oxeltra 5mg modified-release tablets (wockhardt uk ltd) | | 5 | 2 | 10 | PCGP |
| oxycodone | oxeltra 60mg modified-release tablets (wockhardt uk ltd) | | 60 | 2 | 120 | PCGP |
| oxycodone | oxeltra 80mg modified-release tablets (wockhardt uk ltd) | | 80 | 2 | 160 | PCGP |
| oxycodone | oxyact 10mg tablets (kent pharmaceuticals ltd) | | 10 | 2 | 20 | PCGP |
| oxycodone | oxyact 20mg tablets (kent pharmaceuticals ltd) | | 20 | 2 | 40 | PCGP |
| oxycodone | oxyact 5mg tablets (kent pharmaceuticals ltd) | | 5 | 2 | 10 | PCGP |
| oxycodone | oxycodone 10mg capsules | | 10 | 2 | 20 | PCGP |
| oxycodone | oxycodone 10mg modified-release tablets | | 10 | 2 | 20 | PCGP |
| oxycodone | oxycodone 10mg tablets | | 10 | 2 | 20 | PCGP |
| oxycodone | oxycodone 10mg/1ml solution for injection ampoules | | 10 | 3 | 30 | PCGP |
| oxycodone | oxycodone 10mg/ml oral solution sugar free | | 10 | 2 | 20 | PCGP |
| oxycodone | oxycodone 120mg modified-release tablets | | 120 | 2 | 240 | PCGP |
| oxycodone | oxycodone 15mg modified-release tablets | | 15 | 2 | 30 | PCGP |
| oxycodone | oxycodone 20mg capsules | | 20 | 2 | 40 | PCGP |
| oxycodone | oxycodone 20mg modified-release tablets | | 20 | 2 | 40 | PCGP |
| oxycodone | oxycodone 20mg tablets | | 20 | 2 | 40 | PCGP |
| oxycodone | oxycodone 20mg/2ml solution for injection ampoules | | 10 | 3 | 30 | PCGP |
| oxycodone | oxycodone 30mg modified-release tablets | | 30 | 2 | 60 | PCGP |
| oxycodone | oxycodone 40mg modified-release tablets | | 40 | 2 | 80 | PCGP |
| oxycodone | oxycodone 50mg/1ml solution for injection ampoules | | 50 | 3 | 150 | PCGP |
| oxycodone | oxycodone 5mg capsules | | 5 | 2 | 10 | PCGP |
| oxycodone | oxycodone 5mg modified-release tablets | | 5 | 2 | 10 | PCGP |
| oxycodone | oxycodone 5mg tablets | | 5 | 2 | 10 | PCGP |
| oxycodone | oxycodone 5mg/5ml oral solution sugar free | | 5 | 2 | 10 | PCGP |
| oxycodone | oxycodone 60mg modified-release tablets | | 60 | 2 | 120 | PCGP |
| oxycodone | oxycodone 80mg modified-release tablets | | 80 | 2 | 160 | PCGP |
| oxycodone | oxycontin 10mg modified-release tablets (napp pharmaceuticals ltd) | | 10 | 2 | 20 | PCGP |
| oxycodone | oxycontin 120mg modified-release tablets (napp pharmaceuticals ltd) | | 120 | 2 | 240 | PCGP |
| oxycodone | oxycontin 15mg modified-release tablets (napp pharmaceuticals ltd) | | 15 | 2 | 30 | PCGP |
| oxycodone | oxycontin 20mg modified-release tablets (napp pharmaceuticals ltd) | | 20 | 2 | 40 | PCGP |
| oxycodone | oxycontin 30mg modified-release tablets (napp pharmaceuticals ltd) | | 30 | 2 | 60 | PCGP |
| oxycodone | oxycontin 40mg modified-release tablets (napp pharmaceuticals ltd) | | 40 | 2 | 80 | PCGP |
| oxycodone | oxycontin 5mg modified-release tablets (napp pharmaceuticals ltd) | | 5 | 2 | 10 | PCGP |
| oxycodone | oxycontin 60mg modified-release tablets (napp pharmaceuticals ltd) | | 60 | 2 | 120 | PCGP |
| oxycodone | oxycontin 80mg modified-release tablets (napp pharmaceuticals ltd) | | 80 | 2 | 160 | PCGP |
| oxycodone | oxylan 10mg modified-release tablets (healthcare pharma ltd) | | 10 | 2 | 20 | PCGP |
| oxycodone | oxylan 20mg modified-release tablets (healthcare pharma ltd) | | 20 | 2 | 40 | PCGP |
| oxycodone | oxylan 40mg modified-release tablets (healthcare pharma ltd) | | 40 | 2 | 80 | PCGP |
| oxycodone | oxylan 5mg modified-release tablets (healthcare pharma ltd) | | 5 | 2 | 10 | PCGP |
| oxycodone | oxylan 80mg modified-release tablets (healthcare pharma ltd) | | 80 | 2 | 160 | PCGP |
| oxycodone | oxynorm 10mg capsules (napp pharmaceuticals ltd) | | 10 | 2 | 20 | PCGP |
| oxycodone | oxynorm 10mg/1ml solution for injection ampoules (napp pharmaceuticals ltd) | | 10 | 3 | 30 | PCGP |
| oxycodone | oxynorm 10mg/ml concentrate oral solution (napp pharmaceuticals ltd) | | 10 | 2 | 20 | PCGP |
| oxycodone | oxynorm 20mg capsules (napp pharmaceuticals ltd) | | 20 | 2 | 40 | PCGP |
| oxycodone | oxynorm 20mg/2ml solution for injection ampoules (napp pharmaceuticals ltd) | | 10 | 3 | 30 | PCGP |
| oxycodone | oxynorm 50mg/1ml solution for injection ampoules (napp pharmaceuticals ltd) | | 50 | 3 | 150 | PCGP |
| oxycodone | oxynorm 5mg capsules (napp pharmaceuticals ltd) | | 5 | 2 | 10 | PCGP |
| oxycodone | oxynorm liquid 1mg/ml oral solution (napp pharmaceuticals ltd) | | 1 | 2 | 2 | PCGP |
| oxycodone | oxypro 10mg modified-release tablets (ridge pharma ltd) | | 10 | 2 | 20 | PCGP |
| oxycodone | oxypro 15mg modified-release tablets (ridge pharma ltd) | | 15 | 2 | 30 | PCGP |
| oxycodone | oxypro 20mg modified-release tablets (ridge pharma ltd) | | 20 | 2 | 40 | PCGP |
| oxycodone | oxypro 30mg modified-release tablets (ridge pharma ltd) | | 30 | 2 | 60 | PCGP |
| oxycodone | oxypro 40mg modified-release tablets (ridge pharma ltd) | | 40 | 2 | 80 | PCGP |
| oxycodone | oxypro 5mg modified-release tablets (ridge pharma ltd) | | 5 | 2 | 10 | PCGP |
| oxycodone | oxypro 60mg modified-release tablets (ridge pharma ltd) | | 60 | 2 | 120 | PCGP |
| oxycodone | oxypro 80mg modified-release tablets (ridge pharma ltd) | | 80 | 2 | 160 | PCGP |
| oxycodone | reltebon 10mg modified-release tablets (accord healthcare ltd) | | 10 | 2 | 20 | PCGP |
| oxycodone | reltebon 15mg modified-release tablets (accord healthcare ltd) | | 15 | 2 | 30 | PCGP |
| oxycodone | reltebon 20mg modified-release tablets (accord healthcare ltd) | | 20 | 2 | 40 | PCGP |
| oxycodone | reltebon 30mg modified-release tablets (accord healthcare ltd) | | 30 | 2 | 60 | PCGP |
| oxycodone | reltebon 40mg modified-release tablets (accord healthcare ltd) | | 40 | 2 | 80 | PCGP |
| oxycodone | reltebon 5mg modified-release tablets (accord healthcare ltd) | | 5 | 2 | 10 | PCGP |
| oxycodone | reltebon 60mg modified-release tablets (accord healthcare ltd) | | 60 | 2 | 120 | PCGP |
| oxycodone | reltebon 80mg modified-release tablets (accord healthcare ltd) | | 80 | 2 | 160 | PCGP |
| oxycodone | renocontin 10mg modified-release tablets (glenmark pharmaceuticals europe ltd) | | 10 | 2 | 20 | PCGP |
| oxycodone | renocontin 15mg modified-release tablets (glenmark pharmaceuticals europe ltd) | | 15 | 2 | 30 | PCGP |
| oxycodone | renocontin 20mg modified-release tablets (glenmark pharmaceuticals europe ltd) | | 20 | 2 | 40 | PCGP |
| oxycodone | renocontin 30mg modified-release tablets (glenmark pharmaceuticals europe ltd) | | 30 | 2 | 60 | PCGP |
| oxycodone | renocontin 40mg modified-release tablets (glenmark pharmaceuticals europe ltd) | | 40 | 2 | 80 | PCGP |
| oxycodone | renocontin 5mg modified-release tablets (glenmark pharmaceuticals europe ltd) | | 5 | 2 | 10 | PCGP |
| oxycodone | renocontin 60mg modified-release tablets (glenmark pharmaceuticals europe ltd) | | 60 | 2 | 120 | PCGP |
| oxycodone | shortec 10mg capsules (qdem pharmaceuticals ltd) | | 10 | 2 | 20 | PCGP |
| oxycodone | shortec 10mg/1ml solution for injection ampoules (qdem pharmaceuticals ltd) | | 10 | 3 | 30 | PCGP |
| oxycodone | shortec 10mg/ml concentrate oral solution (qdem pharmaceuticals ltd) | | 10 | 2 | 20 | PCGP |
| oxycodone | shortec 20mg capsules (qdem pharmaceuticals ltd) | | 20 | 2 | 40 | PCGP |
| oxycodone | shortec 20mg/2ml solution for injection ampoules (qdem pharmaceuticals ltd) | | 10 | 3 | 30 | PCGP |
| oxycodone | shortec 50mg/1ml solution for injection ampoules (qdem pharmaceuticals ltd) | | 50 | 3 | 150 | PCGP |
| oxycodone | shortec 5mg capsules (qdem pharmaceuticals ltd) | | 5 | 2 | 10 | PCGP |
| oxycodone | shortec liquid 1mg/ml oral solution (qdem pharmaceuticals ltd) | | 1 | 2 | 2 | PCGP |
| oxycodone | zomestine 10mg modified-release tablets (accord healthcare ltd) | | 10 | 2 | 20 | PCGP |
| oxycodone | zomestine 20mg modified-release tablets (accord healthcare ltd) | | 20 | 2 | 40 | PCGP |
| oxycodone | zomestine 40mg modified-release tablets (accord healthcare ltd) | | 40 | 2 | 80 | PCGP |
| oxycodone | zomestine 5mg modified-release tablets (accord healthcare ltd) | | 5 | 2 | 10 | PCGP |
| oxycodone | zomestine 80mg modified-release tablets (accord healthcare ltd) | | 80 | 2 | 160 | PCGP |
| paracetamol/tramadol | tramacet 37.5mg/325mg effervescent tablets (grunenthal ltd) | | 37.5 | 0.1 | 3.75 | PCGP |
| paracetamol/tramadol | tramacet 37.5mg/325mg tablets (grunenthal ltd) | | 37.5 | 0.1 | 3.75 | PCGP |
| paracetamol/tramadol | tramadol 37.5mg / paracetamol 325mg effervescent tablets sugar free | | 37.5 | 0.1 | 3.75 | PCGP |
| paracetamol/tramadol | tramadol 37.5mg / paracetamol 325mg tablets | | 37.5 | 0.1 | 3.75 | PCGP |
| paracetamol/tramadol | tramadol 75mg / paracetamol 650mg tablets | | 75 | 0.1 | 7.5 | PCGP |
| paracetamol/tramadol | trapadex 37.5mg/325mg tablets (noumed life sciences ltd) | | 37.5 | 0.1 | 3.75 | PCGP |
| pentazocine | fortral 25mg tablets (zentiva) | | 25 | 0.37 | 9.25 | OMCF |
| pentazocine | fortral capsules 50 mg | | 50 | 0.37 | 18.5 | OMCF |
| pentazocine | fortral injection 30 mg/ml | | 30 | 0.5 | 15 | UCSF |
| pentazocine | pentazocine 25mg tablets | | 25 | 0.37 | 9.25 | OMCF |
| pentazocine | pentazocine 50mg capsules | | 50 | 0.37 | 18.5 | OMCF |
| pentazocine | pentazocine lactate injection 30 mg/ml | | 30 | 0.5 | 15 | UCSF |
| pentazocine | fortral 30mg/1ml solution for injection ampoules (zentiva) | | 30 | 0.5 | 15 | UCSF |
| pentazocine | fortral 60mg/2ml solution for injection ampoules (zentiva) | | 60 | 0.5 | 30 | UCSF |
| pentazocine | pentazocine 30mg/1ml solution for injection ampoules | | 30 | 0.5 | 15 | UCSF |
| pentazocine | pentazocine 60mg/2ml solution for injection ampoules | | 60 | 0.5 | 30 | UCSF |
| tapentadol | palexia 20mg/ml oral solution (grunenthal ltd) | | 20 | 0.4 | 8 | OMCF |
| tapentadol | palexia 50mg tablets (grunenthal ltd) | | 50 | 0.4 | 20 | OMCF |
| tapentadol | palexia 75mg tablets (grunenthal ltd) | | 75 | 0.4 | 30 | OMCF |
| tapentadol | palexia sr 100mg tablets (grunenthal ltd) | | 100 | 0.4 | 40 | OMCF |
| tapentadol | palexia sr 150mg tablets (grunenthal ltd) | | 150 | 0.4 | 60 | OMCF |
| tapentadol | palexia sr 200mg tablets (grunenthal ltd) | | 200 | 0.4 | 80 | OMCF |
| tapentadol | palexia sr 250mg tablets (grunenthal ltd) | | 250 | 0.4 | 100 | OMCF |
| tapentadol | palexia sr 50mg tablets (grunenthal ltd) | | 50 | 0.4 | 20 | OMCF |
| tapentadol | tapentadol 100mg modified-release tablets | | 100 | 0.4 | 40 | OMCF |
| tapentadol | tapentadol 150mg modified-release tablets | | 150 | 0.4 | 60 | OMCF |
| tapentadol | tapentadol 200mg modified-release tablets | | 200 | 0.4 | 80 | OMCF |
| tapentadol | tapentadol 20mg/ml oral solution sugar free | | 20 | 0.4 | 8 | OMCF |
| tapentadol | tapentadol 250mg modified-release tablets | | 250 | 0.4 | 100 | OMCF |
| tapentadol | tapentadol 50mg modified-release tablets | | 50 | 0.4 | 20 | OMCF |
| tapentadol | tapentadol 50mg tablets | | 50 | 0.4 | 20 | OMCF |
| tapentadol | tapentadol 75mg tablets | | 75 | 0.4 | 30 | OMCF |
| tramadol | brimisol pr 100mg tablets (bristol laboratories ltd) | | 100 | 0.1 | 10 | PCGP |
| tramadol | brimisol pr 200mg tablets (bristol laboratories ltd) | | 200 | 0.1 | 20 | PCGP |
| tramadol | dromadol sr 100mg tablets (teva uk ltd) | | 100 | 0.1 | 10 | PCGP |
| tramadol | dromadol sr 150mg tablets (teva uk ltd) | | 150 | 0.1 | 15 | PCGP |
| tramadol | dromadol sr 200mg tablets (teva uk ltd) | | 200 | 0.1 | 20 | PCGP |
| tramadol | dromadol sr 75mg tablets (ivax pharmaceuticals uk ltd) | | 75 | 0.1 | 7.5 | PCGP |
| tramadol | dromadol xl 150mg tablets (ivax pharmaceuticals uk ltd) | | 150 | 0.1 | 15 | PCGP |
| tramadol | dromadol xl 200mg tablets (ivax pharmaceuticals uk ltd) | | 200 | 0.1 | 20 | PCGP |
| tramadol | dromadol xl 300mg tablets (ivax pharmaceuticals uk ltd) | | 300 | 0.1 | 30 | PCGP |
| tramadol | dromadol xl 400mg tablets (ivax pharmaceuticals uk ltd) | | 400 | 0.1 | 40 | PCGP |
| tramadol | invodol sr 100mg tablets (ennogen healthcare ltd) | | 100 | 0.1 | 10 | PCGP |
| tramadol | invodol sr 150mg tablets (ennogen healthcare ltd) | | 150 | 0.1 | 15 | PCGP |
| tramadol | invodol sr 200mg tablets (ennogen healthcare ltd) | | 200 | 0.1 | 20 | PCGP |
| tramadol | larapam (piroxicam) capsules 10 mg | | 10 | 0.1 | 1 | PCGP |
| tramadol | larapam (piroxicam) capsules 20 mg | | 20 | 0.1 | 2 | PCGP |
| tramadol | larapam sr 100mg tablets (sandoz ltd) | | 100 | 0.1 | 10 | PCGP |
| tramadol | larapam sr 150mg tablets (sandoz ltd) | | 150 | 0.1 | 15 | PCGP |
| tramadol | larapam sr 200mg tablets (sandoz ltd) | | 200 | 0.1 | 20 | PCGP |
| tramadol | mabron 100mg modified-release tablets (teva uk ltd) | | 100 | 0.1 | 10 | PCGP |
| tramadol | mabron 150mg modified-release tablets (teva uk ltd) | | 150 | 0.1 | 15 | PCGP |
| tramadol | mabron 200mg modified-release tablets (teva uk ltd) | | 200 | 0.1 | 20 | PCGP |
| tramadol | maneo 100mg modified-release tablets (mylan) | | 100 | 0.1 | 10 | PCGP |
| tramadol | maneo 150mg modified-release tablets (mylan) | | 150 | 0.1 | 15 | PCGP |
| tramadol | maneo 200mg modified-release tablets (mylan) | | 200 | 0.1 | 20 | PCGP |
| tramadol | marol 100mg modified-release tablets (teva uk ltd) | | 100 | 0.1 | 10 | PCGP |
| tramadol | marol 150mg modified-release tablets (teva uk ltd) | | 150 | 0.1 | 15 | PCGP |
| tramadol | marol 200mg modified-release tablets (teva uk ltd) | | 200 | 0.1 | 20 | PCGP |
| tramadol | maxitram sr 100mg capsules (chiesi ltd) | | 100 | 0.1 | 10 | PCGP |
| tramadol | maxitram sr 150mg capsules (chiesi ltd) | | 150 | 0.1 | 15 | PCGP |
| tramadol | maxitram sr 200mg capsules (chiesi ltd) | | 200 | 0.1 | 20 | PCGP |
| tramadol | maxitram sr 50mg capsules (chiesi ltd) | | 50 | 0.1 | 5 | PCGP |
| tramadol | nobligan retard 100mg tablets (grunenthal ltd) | | 100 | 0.1 | 10 | PCGP |
| tramadol | oldaram 100mg modified-release tablets (ranbaxy (uk) ltd) | | 100 | 0.1 | 10 | PCGP |
| tramadol | oldaram 150mg modified-release tablets (ranbaxy (uk) ltd) | | 150 | 0.1 | 15 | PCGP |
| tramadol | oldaram 200mg modified-release tablets (ranbaxy (uk) ltd) | | 200 | 0.1 | 20 | PCGP |
| tramadol | tilodol sr 100mg tablets (sandoz ltd) | | 100 | 0.1 | 10 | PCGP |
| tramadol | tilodol sr 150mg tablets (sandoz ltd) | | 150 | 0.1 | 15 | PCGP |
| tramadol | tilodol sr 200mg tablets (sandoz ltd) | | 200 | 0.1 | 20 | PCGP |
| tramadol | tradorec xl 100mg tablets (endo ventures ltd) | | 100 | 0.1 | 10 | PCGP |
| tramadol | tradorec xl 200mg tablets (endo ventures ltd) | | 200 | 0.1 | 20 | PCGP |
| tramadol | tradorec xl 300mg tablets (endo ventures ltd) | | 300 | 0.1 | 30 | PCGP |
| tramadol | tramadol 100mg effervescent powder sachets sugar free | | 100 | 0.1 | 10 | PCGP |
| tramadol | tramadol 100mg modified-release capsules | | 100 | 0.1 | 10 | PCGP |
| tramadol | tramadol 100mg modified-release tablets | | 100 | 0.1 | 10 | PCGP |
| tramadol | tramadol 100mg/2ml solution for injection ampoules | | 50 | 0.2 | 5 | OC |
| tramadol | tramadol 100mg/ml oral drops | | 100 | 0.1 | 10 | PCGP |
| tramadol | tramadol 150mg modified-release capsules | | 150 | 0.1 | 15 | PCGP |
| tramadol | tramadol 150mg modified-release tablets | | 150 | 0.1 | 15 | PCGP |
| tramadol | tramadol 200mg modified-release capsules | | 200 | 0.1 | 20 | PCGP |
| tramadol | tramadol 200mg modified-release tablets | | 200 | 0.1 | 20 | PCGP |
| tramadol | tramadol 300mg modified-release tablets | | 300 | 0.1 | 30 | PCGP |
| tramadol | tramadol 400mg modified-release tablets | | 400 | 0.1 | 40 | PCGP |
| tramadol | tramadol 50mg capsules | | 50 | 0.1 | 5 | PCGP |
| tramadol | tramadol 50mg effervescent powder sachets sugar free | | 50 | 0.1 | 5 | PCGP |
| tramadol | tramadol 50mg modified-release capsules | | 50 | 0.1 | 5 | PCGP |
| tramadol | tramadol 50mg modified-release tablets | | 50 | 0.1 | 5 | PCGP |
| tramadol | tramadol 50mg orodispersible tablets sugar free | | 50 | 0.1 | 5 | PCGP |
| tramadol | tramadol 50mg soluble tablets sugar free | | 50 | 0.1 | 5 | PCGP |
| tramadol | tramadol 75mg modified-release tablets | | 75 | 0.1 | 7.5 | PCGP |
| tramadol | tramake 50mg capsules (galen ltd) | | 50 | 0.1 | 5 | PCGP |
| tramadol | tramake insts 100mg sachets (galen ltd) | | 100 | 0.1 | 10 | PCGP |
| tramadol | tramake insts 50mg sachets (galen ltd) | | 50 | 0.1 | 5 | PCGP |
| tramadol | tramquel sr 100mg capsules (mylan) | | 100 | 0.1 | 10 | PCGP |
| tramadol | tramquel sr 150mg capsules (mylan) | | 150 | 0.1 | 15 | PCGP |
| tramadol | tramquel sr 200mg capsules (mylan) | | 200 | 0.1 | 20 | PCGP |
| tramadol | tramquel sr 50mg capsules (mylan) | | 50 | 0.1 | 5 | PCGP |
| tramadol | tramulief sr 100mg tablets (advanz pharma) | | 100 | 0.1 | 10 | PCGP |
| tramadol | tramulief sr 150mg tablets (advanz pharma) | | 150 | 0.1 | 15 | PCGP |
| tramadol | tramulief sr 200mg tablets (advanz pharma) | | 200 | 0.1 | 20 | PCGP |
| tramadol | zamadol 100mg/2ml solution for injection ampoules (mylan) | | 50 | 0.2 | 5 | OC |
| tramadol | zamadol 24hr 150mg modified-release tablets (mylan) | | 150 | 0.1 | 15 | PCGP |
| tramadol | zamadol 24hr 200mg modified-release tablets (mylan) | | 200 | 0.1 | 20 | PCGP |
| tramadol | zamadol 24hr 300mg modified-release tablets (mylan) | | 300 | 0.1 | 30 | PCGP |
| tramadol | zamadol 24hr 400mg modified-release tablets (mylan) | | 400 | 0.1 | 40 | PCGP |
| tramadol | zamadol 50mg capsules (mylan) | | 50 | 0.1 | 5 | PCGP |
| tramadol | zamadol melt 50mg tablets (mylan) | | 50 | 0.1 | 5 | PCGP |
| tramadol | zamadol sr 100mg capsules (mylan) | | 100 | 0.1 | 10 | PCGP |
| tramadol | zamadol sr 150mg capsules (mylan) | | 150 | 0.1 | 15 | PCGP |
| tramadol | zamadol sr 200mg capsules (mylan) | | 200 | 0.1 | 20 | PCGP |
| tramadol | zamadol sr 50mg capsules (mylan) | | 50 | 0.1 | 5 | PCGP |
| tramadol | zeridame sr 100mg tablets (actavis uk ltd) | | 100 | 0.1 | 10 | PCGP |
| tramadol | zeridame sr 150mg tablets (actavis uk ltd) | | 150 | 0.1 | 15 | PCGP |
| tramadol | zeridame sr 200mg tablets (actavis uk ltd) | | 200 | 0.1 | 20 | PCGP |
| tramadol | zydol 100mg/2ml solution for injection ampoules (grunenthal ltd) | | 50 | 0.2 | 5 | OC |
| tramadol | zydol 50mg capsules (grunenthal ltd) | | 50 | 0.1 | 5 | PCGP |
| tramadol | zydol 50mg soluble tablets (grunenthal ltd) | | 50 | 0.1 | 5 | PCGP |
| tramadol | zydol sr 100mg tablets (grunenthal ltd) | | 100 | 0.1 | 10 | PCGP |
| tramadol | zydol sr 150mg tablets (grunenthal ltd) | | 150 | 0.1 | 15 | PCGP |
| tramadol | zydol sr 200mg tablets (grunenthal ltd) | | 200 | 0.1 | 20 | PCGP |
| tramadol | zydol sr 50mg tablets (grunenthal ltd) | | 50 | 0.1 | 5 | PCGP |
| tramadol | zydol xl 150mg tablets (grunenthal ltd) | | 150 | 0.1 | 15 | PCGP |
| tramadol | zydol xl 200mg tablets (grunenthal ltd) | | 200 | 0.1 | 20 | PCGP |
| tramadol | zydol xl 300mg tablets (grunenthal ltd) | | 300 | 0.1 | 30 | PCGP |
| tramadol | zydol xl 400mg tablets (grunenthal ltd) | | 400 | 0.1 | 40 | PCGP |
| tramadol | zytram sr 100mg tablets (qdem pharmaceuticals ltd) | | 100 | 0.1 | 10 | PCGP |
| tramadol | zytram sr 150mg tablets (qdem pharmaceuticals ltd) | | 150 | 0.1 | 15 | PCGP |
| tramadol | zytram sr 200mg tablets (qdem pharmaceuticals ltd) | | 200 | 0.1 | 20 | PCGP |
| tramadol | zytram sr 75mg tablets (qdem pharmaceuticals ltd) | | 75 | 0.1 | 7.5 | PCGP |

**Appendix 2.** Opioid conversion source link

| **Link to sources** | **Abbreviation** |
| --- | --- |
| https://book.pallcare.info/index.php?op=plugin&src=opiconv | PCGP |
| https://pain.ucsf.edu/opioid-analgesics/calculation-oral-morphine-equivalents-ome | UCSF |
| https://www.ncbi.nlm.nih.gov/pmc/articles/PMC3217286/#:~:text=Thus%2C%20orodispersible%20tablets%20are%20solid,without%20any%20difficulty%20of%20swallowing. | OD |
| https://www.nhsbsa.nhs.uk/access-our-data-products/epact2/dashboards-and-specifications/opioid-prescribing-comparators-dashboard (Section 2.2) | OPM |
| https://www.hhs.gov/guidance/sites/default/files/hhs-guidance-documents/Opioid%20Morphine%20EQ%20Conversion%20Factors%20%28vFeb%202018%29.pdf | OMCF |
| https://bnf.nice.org.uk/medicines-guidance/prescribing-in-palliative-care/?UNLID= | NICE |
| https://www.eviq.org.au/clinical-resources/eviq-calculators/3201-opioid-conversion-calculator | OC |

**Table** **S1:** Characteristics of patients who were prescribed opioids by ethnic group

| Variables | **Overall** | **Black** | **Mixed** | **Other** | **South Asian** | **White** |
| --- | --- | --- | --- | --- | --- | --- |
| n | 183646 | 3615 | 815 | 1210 | 3554 | 174452 |
| Age (mean (SD)) | 76·60 (12·26) | 72·20 (14·44) | 71·86 (14·45) | 70·98 (15·00) | 71·53 (13·74) | 76·86 (12·10) |
| Age group (%) |  |  |  |  |  |  |
| 18-50 | 5804 ( 3·2) | 314 ( 8·7) | 74 ( 9·1) | 130 (10·7) | 294 ( 8·3) | 4992 ( 2·9) |
| 50-59 | 13890 ( 7·6) | 564 (15·6) | 118 (14·5) | 170 (14·0) | 442 (12·4) | 12596 (7·2) |
| 60-69 | 32013 (17·4) | 527 (14·6) | 142 (17·4) | 247 (20·4) | 777 (21·9) | 30320 (17·4) |
| 70-79 | 53649 (29·2) | 925 (25·6) | 215 (26·4) | 279 (23·1) | 965 (27·2) | 51265 (29·4) |
| 80+ | 78290 (42·6) | 1285 (35·5) | 266 (32·6) | 384 (31·7) | 1076 (30·3) | 75279 (43·2) |
| sex (%) |  |  |  |  |  |  |
| Female | 87899 (47·9) | 1468 (40·6) | 365 (44·8) | 560 (46·3) | 1615 (45·4) | 83891 (48·1) |
| Male | 95747 (52·1) | 2147 (59·4) | 450 (55·2) | 650 (53·7) | 1939 (54·6) | 90561 (51·9) |
| Cancer Site (%) |  |  |  |  |  |  |
| Bone | 3073 (1·7) | 50 (1·4) | 14 (1·7) | 27 (2·2) | 57 (1·6) | 2925 (1·7) |
| Multiple myeloma | 4465 (2·4) | 222 (6·1) | 47 (5·8) | 30 (2·5) | 119 (3·3) | 4047 (2·3) |
| Breast | 32852 (17·9) | 550 (15·2) | 119 (14·6) | 175 (14·5) | 652 (18·3) | 31356 (18·0) |
| Cervix | 2247 (1·2) | 49 (1·4) | 8 (1·0) | 23 (1·9) | 41 (1·2) | 2126 (1·2) |
| Colorectal | 33353 (18·2) | 514 (14·2) | 132 (16·2) | 193 (16·0) | 548 (15·4) | 31966 (18·3) |
| Head and neck | 7033 (3·8) | 90 (2·5) | 30 (3·7) | 45 (3·7) | 174 (4·9) | 6694 (3·8) |
| Liver | 7146 (3·9) | 139 (3·8) | 34 (4·2) | 87 (7·2) | 271 (7·6) | 6615 (3·8) |
| Lung | 47687 (26·0) | 649 (18·0) | 196 (24·0) | 343 (28·3) | 860 (24·2) | 45639 (26·2) |
| Pancreas | 10217 (5·6) | 218 (6·0) | 55 (6·7) | 78 (6·4) | 231 (6·5) | 9635 (5·5) |
| Prostate | 30348 (16·5) | 956 (26·4) | 146 (17·9) | 154 (12·7) | 455 (12·8) | 28637 (16·4) |
| Stomach | 5225 (2·8) | 178 (4·9) | 34 (4·2) | 55 (4·5) | 146 (4·1) | 4812 (2·8) |
| Palliative Care QOF (%) |  |  |  |  |  |  |
| No | 72789 (39·6) | 1671 (46·2) | 305 (37·4) | 479 (39·6) | 1465 (41·2) | 68869 (39·5) |
| Yes | 110857 (60·4) | 1944 (53·8) | 510 (62·6) | 731 (60·4) | 2089 (58·8) | 105583 (60·5) |
| Comorbidity counts (%) |  |  |  |  |  |  |
| Missing | 324 (0·2) | 17 (0·5) | 3 (0·4) | 5 (0·4) | 15 (0·4) | 284 (0·2) |
| 0 | 22369 (12·2) | 516 (14·3) | 136 (16·7) | 245 (20·2) | 459 (12·9) | 21013 (12·0) |
| 1 | 37899 (20·6) | 766 (21·2) | 138 (16·9) | 305 (25·2) | 628 (17·7) | 36062 (20·7) |
| 2 | 40522 (22·1) | 808 (22·4) | 172 (21·1) | 227 (18·8) | 728 (20·5) | 38587 (22·1) |
| 3 | 34190 (18·6) | 700 (19·4) | 165 (20·2) | 168 (13·9) | 666 (18·7) | 32491 (18·6) |
| 4 | 23824 (13·0) | 386 (10·7) | 86 (10·6) | 130 (10·7) | 512 (14·4) | 22710 (13·0) |
| 5+ | 24518 (13·4) | 422 (11·7) | 115 (14·1) | 130 (10·7) | 546 (15·4) | 23305 (13·4) |
| Practice level Urban-rural (%) |  |  |  |  |  |  |
| Missing | 170 (0·1) | 3 (0·1) | 0 (0·0) | 3 (0·2) | 5 (0·1) | 159 (0·1) |
| Rural | 26836 (14·6) | 22 (0·6) | 35 (4·3) | 61 (5·0) | 38 (1·1) | 26680 (15·3) |
| Urban | 156640 (85·3) | 3590 (99·3) | 780 (95·7) | 1146 (94·7) | 3511 (98·8) | 147613 (84·6) |
| Practice region (%) |  |  |  |  |  |  |
| East Midlands | 3449 (1·9) | 61 (1·7) | 7 (0·9) | 14 (1·2) | 53 (1·5) | 3314 (1·9) |
| East of England | 7831 (4·3) | 46 (1·3) | 25 (3·1) | 50 (4·1) | 95 (2·7) | 7615 (4·4) |
| London | 24150 (13·2) | 170 (4·7) | 49 (6·0) | 84 (6·9) | 95 (2·7) | 23752 (13·6) |
| North East | 8108 (4·4) | 6 (0·2) | 8 (1·0) | 12 (1·0) | 23 (0·6) | 8059 (4·6) |
| North West | 40090 (21·8) | 181 (5·0) | 85 (10·4) | 142 (11·7) | 407 (11·5) | 39275 (22·5) |
| South Central | 37573 (20·5) | 208 (5·8) | 112 (13·7) | 215 (17·8) | 367 (10·3) | 36671 (21·0) |
| South West | 22845 (12·4) | 2500 (69·2) | 413 (50·7) | 560 (46·3) | 1752 (49·3) | 17620 (10·1) |
| West Midlands | 32309 (17·6) | 423 (11·7) | 108 (13·3) | 121 (10·0) | 725 (20·4) | 30932 (17·7) |
| Yorkshire & The Humber | 7291 (4·0) | 20 (0·6) | 8 (1·0) | 12 (1·0) | 37 (1·0) | 7214 (4·1) |
| IMD (%) |  |  |  |  |  |  |
| 1 | 30449 (16·6) | 136 (3·8) | 104 (12·8) | 193 (16·0) | 306 (8·6) | 29710 (17·0) |
| 2 | 31588 (17·2) | 196 (5·4) | 73 (9·0) | 161 (13·3) | 426 (12·0) | 30732 (17·6) |
| 3 | 38358 (20·9) | 656 (18·2) | 157 (19·3) | 258 (21·4) | 660 (18·6) | 36627 (21·0) |
| 4 | 38409 (20·9) | 1224 (33·9) | 249 (30·6) | 327 (27·1) | 1017 (28·7) | 35592 (20·4) |
| 5 | 44671 (24·3) | 1400 (38·8) | 232 (28·5) | 268 (22·2) | 1139 (32·1) | 41632 (23·9) |
| Opioid Prescription |  |  |  |  |  |  |
| Total Opioid prescription | 3987635 | 67096 | 13784 | 21050 | 68416 | 3817289 |
| Opioid Prescription (mean (SD)) | 21·71 (37·54) | 18·56 (31·40) | 16·91 (28·40) | 17·40 (30·12) | 19·25 (32·99) | 21·88 (37·82) |

Data are n (%), unless otherwise specified. *Values are in the final three months of life· QOF: Quality of Outcome Framework. IMD=Index for Multiple Deprivation

**Table S2**: Characteristics of individuals prescribed opioids by ethnic group in the final three months of life

| level | Overall | Black | Mixed | Other | South Asian | White |
| --- | --- | --- | --- | --- | --- | --- |
| n | 124639 | 2066 | 500 | 783 | 2064 | 119226 |
| Age (mean (SD)) | 75·44 (12·40) | 70·48 (14·62) | 70·29 (14·84) | 69·44 (15·28) | 69·70 (13·73) | 75·68 (12·24) |
| Age group (%) |  |  |  |  |  |  |
| 18-50 | 4556 (3·7) | 214 (10·4) | 53 (10·6) | 98 (12·5) | 200 ( 9·7) | 3991 ( 3·3) |
| 50-59 | 10709 (8·6) | 364 (17·6) | 85 (17·0) | 123 (15·7) | 302 (14·6) | 9835 ( 8·2) |
| 60-69 | 24217 (19·4) | 320 (15·5) | 86 (17·2) | 168 (21·5) | 492 (23·8) | 23151 (19·4) |
| 70-79 | 37365 (30·0) | 534 (25·8) | 134 (26·8) | 179 (22·9) | 570 (27·6) | 35948 (30·2) |
| 80+ | 47792 (38·3) | 634 (30·7) | 142 (28·4) | 215 (27·5) | 500 (24·2) | 46301 (38·8) |
| Sex (%) |  |  |  |  |  |  |
| Female | 59645 (47·9) | 852 (41·2) | 225 (45·0) | 373 (47·6) | 939 (45·5) | 57256 (48·0) |
| Male | 64994 (52·1) | 1214 (58·8) | 275 (55·0) | 410 (52·4) | 1125 (54·5) | 61970 (52·0) |
| Cancer site (%) |  |  |  |  |  |  |
| Bone | 2243 (1·8) | 30 (1·5) | 10 (2·0) | 20 (2·6) | 38 (1·8) | 2145 (1·8) |
| Multiple Myeloma | 2564 (2·1) | 100 (4·8) | 22 (4·4) | 15 (1·9) | 59 (2·9) | 2368 (2·0) |
| Breast | 20778 (16·7) | 309 (15·0) | 71 (14·2) | 113 (14·4) | 375 (18·2) | 19910 (16·7) |
| Cervix | 1487 (1·2) | 35 (1·7) | 6 (1·2) | 15 (1·9) | 25 (1·2) | 1406 (1·2) |
| Colorectal | 22474 (18·0) | 331 (16·0) | 88 (17·6) | 120 (15·3) | 327 (15·8) | 21608 (18·1) |
| Head & neck | 4618 (3·7) | 50 (2·4) | 17 (3·4) | 24 (3·1) | 112 (5·4) | 4415 (3·7) |
| Liver | 4572 (3·7) | 76 (3·7) | 21 (4·2) | 62 (7·9) | 168 ( 8·1) | 4245 (3·6) |
| Lung | 35124 (28·2) | 390 (18·9) | 136 (27·2) | 217 (27·7) | 474 (23·0) | 33907 (28·4) |
| Pancreas | 8052 (6·5) | 147 (7·1) | 38 (7·6) | 62 (7·9) | 151 (7·3) | 7654 (6·4) |
| Prostate | 18811 (15·1) | 482 (23·3) | 71 (14·2) | 93 (11·9) | 237 (11·5) | 17928 (15·0) |
| Stomach | 3916 (3·1) | 116 (5·6) | 20 (4·0) | 42 (5·4) | 98 (4·7) | 3640 (3·1) |
| Palliative care QOF (%) |  |  |  |  |  |  |
| No | 41148 (33·0) | 727 (35·2) | 146 (29·2) | 246 (31·4) | 647 (31·3) | 39382 (33·0) |
| Yes | 83491 (67·0) | 1339 (64·8) | 354 (70·8) | 537 (68·6) | 1417 (68·7) | 79844 (67·0) |
| Comorbidity counts (%) |  |  |  |  |  |  |
| Missing | 188 (0·2) | 6 (0·3) | 1 (0·2) | 4 (0·5) | 6 (0·3) | 171 (0·1) |
| 0 | 17768 (14·3) | 344 (16·7) | 107 (21·4) | 187 (23·9) | 325 (15·7) | 16805 (14·1) |
| 1 | 28194 (22·6) | 490 (23·7) | 95 (19·0) | 209 (26·7) | 429 (20·8) | 26971 (22·6) |
| 2 | 28346 (22·7) | 466 (22·6) | 104 (20·8) | 146 (18·6) | 454 (22·0) | 27176 (22·8) |
| 3 | 22066 (17·7) | 372 (18·0) | 92 (18·4) | 100 (12·8) | 353 (17·1) | 21149 (17·7) |
| 4 | 14486 (11·6) | 186 (9·0) | 47 (9·4) | 73 (9·3) | 265 (12·8) | 13915 (11·7) |
| 5+ | 13591 (10·9) | 202 (9·8) | 54 (10·8) | 64 (8·2) | 232 (11·2) | 13039 (10·9) |
| Practice level urban-rural (%) |  |  |  |  |  |  |
|  | 125 (0·1) | 3 (0·1) | 0 (0·0) | 3 (0·4) | 5 (0·2) | 114 (0·1) |
| Rural | 18782 (15·1) | 13 (0·6) | 27 (5·4) | 42 (5·4) | 27 (1·3) | 18673 (15·7) |
| Urban | 105732 (84·8) | 2050 (99·2) | 473 (94·6) | 738 (94·3) | 2032 (98·4) | 100439 (84·2) |
| Practice Region (%) |  |  |  |  |  |  |
| East Midlands | 2375 (1·9) | 41 (2·0) | 7 (1·4) | 7 (0·9) | 33 (1·6) | 2287 (1·9) |
| East of England | 5468 (4·4) | 29 (1·4) | 15 (3·0) | 36 (4·6) | 57 (2·8) | 5331 (4·5) |
| London | 17091 (13·7) | 120 (5·8) | 34 (6·8) | 56 (7·2) | 65 (3·1) | 16816 (14·1) |
| North East | 5605 (4·5) | 2 (0·1) | 6 (1·2) | 8 (1·0) | 17 (0·8) | 5572 (4·7) |
| North West | 27497 (22·1) | 118 (5·7) | 54 (10·8) | 94 (12·0) | 243 (11·8) | 26988 (22·6) |
| South Central | 25652 (20·6) | 128 (6·2) | 71 (14·2) | 144 (18·4) | 226 (10·9) | 25083 (21·0) |
| South West | 14006 (11·2) | 1375 (66·6) | 231 (46·2) | 345 (44·1) | 954 (46·2) | 11101 (9·3) |
| West Midlands | 21758 (17·5) | 239 (11·6) | 77 (15·4) | 85 (10·9) | 449 (21·8) | 20908 (17·5) |
| Yorkshire & The Humber | 5187 (4·2) | 14 (0·7) | 5 (1·0) | 8 (1·0) | 20 (1·0) | 5140 (4·3) |
| IMD quintile (%) |  |  |  |  |  |  |
| 1 | 20864 (16·8) | 78 (3·8) | 64 (12·8) | 125 (16·0) | 181 (8·8) | 20416 (17·1) |
| 2 | 21610 (17·4) | 106 (5·1) | 47 (9·4) | 111 (14·2) | 261 (12·7) | 21085 (17·7) |
| 3 | 26129 (21·0) | 378 (18·3) | 102 (20·4) | 179 (22·9) | 392 (19·0) | 25078 (21·1) |
| 4 | 25711 (20·6) | 688 (33·3) | 146 (29·2) | 192 (24·6) | 537 (26·1) | 24148 (20·3) |
| 5 | 30199 (24·3) | 813 (39·4) | 141 (28·2) | 173 (22·2) | 687 (33·4) | 28385 (23·8) |
| Opioid Prescription |  |  |  |  |  |  |
| Total | 620232 | 9161 | 2186 | 3702 | 9649 | 595534 |
| Opioid Prescription (mean (SD)) | 4·98 (4·63) | 4·43 (4·30) | 4·37 (3·86) | 4·73 (4·36) | 4·67 (4·31) | 5·00 (4·64) |

Data are n (%), unless otherwise specified. *Values are in the final three months of life· QOF: Quality of Outcome Framework. IMD=Index for Multiple Deprivation

**Table S3:** Association between patient ethnicity and opioid prescribing (16 ethnic subgroups)

| Ethnicity | Adjusted Rate Ratios (aRRs) | Lower CI | Upper CI | P value |
| --- | --- | --- | --- | --- |
| African | 0.82 | 0.75 | 0.90 | 0.00 |
| Bangladeshi | 0.93 | 0.81 | 1.08 | 0.36 |
| Caribbean | 0.95 | 0.89 | 1.01 | 0.08 |
| Chinese | 0.90 | 0.78 | 1.05 | 0.19 |
| Indian | 0.94 | 0.89 | 1.00 | 0.05 |
| Irish | 1.09 | 1.04 | 1.14 | 0.00 |
| Other Asian | 0.95 | 0.86 | 1.04 | 0.24 |
| Other Black | 0.97 | 0.84 | 1.11 | 0.63 |
| Other ethnic group | 0.91 | 0.84 | 1.00 | 0.04 |
| Other Mixed | 0.84 | 0.71 | 0.99 | 0.04 |
| Other White | 1.00 | 0.97 | 1.03 | 0.83 |
| Pakistani | 0.89 | 0.82 | 0.97 | 0.01 |
| Unknown/Not Stated | 0.98 | 0.97 | 1.00 | 0.01 |
| White and Asian | 0.83 | 0.63 | 1.08 | 0.16 |
| White and Black African | 0.78 | 0.64 | 0.95 | 0.01 |
| White and Black Caribbean | 0.90 | 0.80 | 1.01 | 0.08 |

Data are n (%), unless otherwise specified . White British is the Reference category* CI: Confidence Interval

**Table S4:** Characteristics of individuals who were admitted to hospital by ethnic group between 2011 to 2019

|  | Overall | Black | Mixed | Other | South Asian | White |
| --- | --- | --- | --- | --- | --- | --- |
| n | 201863 | 3879 | 891 | 1309 | 3647 | 192137 |
| Age (mean (SD)) | 77·02 (12·2) | 72·32 (14·3) | 71·8 (14·6) | 71·38 (15·2) | 71·60 (13·8) | 77·28 (12·0) |
| Age group (%) |  |  |  |  |  |  |
| 18-50 | 6055 (3·0) | 343 (8·8) | 81 (9·1) | 143 (10·9) | 306 (8·4) | 5182 (2·7) |
| 50-59 | 14396 (7·1) | 580 (15·0) | 133 (14·9) | 172 (13·1) | 453 (12·4) | 13058 (6·8) |
| 60-69 | 34067 (16·9) | 553 (14·3) | 148 (16·6) | 265 (20·2) | 775 (21·3) | 32326 (16·8) |
| 70-79 | 58161 (28·8) | 1005 (25·9) | 246 (27·6) | 300 (22·9) | 1006 (27·6) | 55604 (28·9) |
| 80+ | 89184 (44·2) | 1398 (36·0) | 283 (31·8) | 429 (32·8) | 1107 (30·4) | 85967 (44·7) |
| Sex (%) |  |  |  |  |  |  |
| Female | 94046 (46·6) | 1498 (38·6) | 393 (44·1) | 612 (46·8) | 1620 (44·4) | 89923 (46·8) |
| Male | 107817 (53·4) | 2381 (61·4) | 498 (55·9) | 697 (53·2) | 2027 (55·6) | 102214 (53·2) |
| Practice level urban rural (%) |  |  |  |  |  |  |
| Missing | 179 (0·1) | 3 (0·1) | 0 (0·0) | 3 (0·2) | 5 (0·1) | 168 (0·1) |
| Rural | 29145 (14·4) | 20 (0·5) | 35 (3·9) | 65 (5·0) | 40 (1·1) | 28985 (15·1) |
| Urban | 172539 (85·5) | 3856 (99·4) | 856 (96·1) | 1241 (94·8) | 3602 (98·8) | 162984 (84·8) |
| Practice Region (%) |  |  |  |  |  |  |
| East Midlands | 3764 (1·9) | 57 (1·5) | 7 (0·8) | 11 (0·8) | 57 (1·6) | 3632 (1·9) |
| East of England | 8856 (4·4) | 51 (1·3) | 24 ( 2·7) | 54 (4·1) | 102 (2·8) | 8625 (4·5) |
| London | 25916 (12·8) | 182 (4·7) | 47 (5·3) | 87 (6·6) | 95 (2·6) | 25505 (13·3) |
| North East | 8769 (4·3) | 6 (0·2) | 10 (1·1) | 14 (1·1) | 22 (0·6) | 8717 (4·5) |
| North West | 43624 (21·6) | 202 (5·2) | 89 (10·0) | 154 (11·8) | 376 (10·3) | 42803 (22·3) |
| South Central | 41517 (20·6) | 215 (5·5) | 116 (13·0) | 236 (18·0) | 374 (10·3) | 40576 (21·1) |
| South West | 26272 (13·0) | 2721 (70·1) | 473 (53·1) | 610 (46·6) | 1865 (51·1) | 20603 (10·7) |
| West Midlands | 35236 (17·5) | 421 (10·9) | 117 (13·1) | 132 (10·1) | 717 (19·7) | 33849 (17·6) |
| Yorkshire & The Humber | 7909 (3·9) | 24 (0·6) | 8 (0·9) | 11 (0·8) | 39 (1·1) | 7827 (4·1) |
| Cancer primary site (%) |  |  |  |  |  |  |
| Bone | 3102 (1·5) | 46 (1·2) | 16 (1·8) | 29 (2·2) | 55 (1·5) | 2956 (1·5) |
| Multiple myeloma | 4882 (2·4) | 248 (6·4) | 45 (5·1) | 27 (2·1) | 132 (3·6) | 4430 (2·3) |
| Breast | 36502 (18·1) | 573 (14·8) | 141 (15·8) | 209 (16·0) | 685 (18·8) | 34894 (18·2) |
| Cervix | 2359 (1·2) | 51 (1·3) | 10 (1·1) | 31 (2·4) | 36 (1·0) | 2231 (1·2) |
| Colorectal | 37983 (18·8) | 557 (14·4) | 145 (16·3) | 219 (16·7) | 573 (15·7) | 36489 (19·0) |
| Head & neck | 7894 (3·9) | 89 (2·3) | 29 (3·3) | 46 (3·5) | 187 (5·1) | 7543 (3·9) |
| Liver | 7361 (3·6) | 144 (3·7) | 41 (4·6) | 93 7·1) | 280 (7·7) | 6803 (3·5) |
| Lung | 49778 (24·7) | 679 (17·5) | 213 (23·9) | 341 (26·1) | 846 (23·2) | 47699 (24·8) |
| Pancreas | 10645 (5·3) | 234 (6·0) | 51 (5·7) | 80 (6·1) | 223 (6·1) | 10057 (5·2) |
| Prostate | 35379 (17·5) | 1052 (27·1) | 167 (18·7) | 174 (13·3) | 487 (13·4) | 33499 (17·4) |
| Stomach | 5978 (3·0) | 206 (5·3) | 33 (3·7) | 60 (4·6) | 143 (3·9) | 5536 (2·9) |
| Palliative care need (%) |  |  |  |  |  |  |
| No | 90160 (44·7) | 1886 (48·6) | 372 (41·8) | 566 (43·2) | 1632 (44·7) | 85704 (44·6) |
| Yes | 111703 (55·3) | 1993 (51·4) | 519 (58·2) | 743 (56·8) | 2015 (55·3) | 106433 (55·4) |
| Comorbidity counts (%) |  |  |  |  |  |  |
| Missing | 404 (0·2) | 15 (0·4) | 1 (0·1) | 3 (0·2) | 19 (0·5) | 366 (0·2) |
| 0 | 25013 (12·4) | 557 (14·4) | 148 (16·6) | 276 (21·1) | 459 (12·6) | 23573 (12·3) |
| 1 | 42192 (20·9) | 846 (21·8) | 169 (19·0) | 347 (26·5) | 669 (18·3) | 40161 (20·9) |
| 2 | 44831 (22·2) | 847 (21·8) | 187 (21·0) | 238 (18·2) | 765 (21·0) | 42794 (22·3) |
| 3 | 37642 (18·6) | 735 (18·9) | 175 (19·6) | 174 (13·3) | 683 (18·7) | 35875 (18·7) |
| 4 | 25915 (12·8) | 427 (11·0) | 93 (10·4) | 140 (10·7) | 533 (14·6) | 24722 (12·9) |
| 5+ | 25866 (12·8) | 452 (11·7) | 118 (13·2) | 131 (10·0) | 519 (14·2) | 24646 (12·8) |
| IMD quintile (%) |  |  |  |  |  |  |
| 1 | 33898 (16·8) | 144 (3·7) | 112 (12·6) | 195 (14·9) | 333 (9·1) | 33114 (17·2) |
| 2 | 35207 (17·5) | 208 (5·4) | 88 (9·9) | 179 (13·7) | 435 (12·0) | 34297 (17·9) |
| 3 | 42021 (20·8) | 699 (18·0) | 170 (19·1) | 296 (22·7) | 688 (18·9) | 40168 (20·9) |
| 4 | 42070 (20·9) | 1333 (34·4) | 283 (31·8) | 351 (26·9) | 1072 (29·5) | 39031 (20·3) |
| 5 | 48486 (24·0) | 1492 (38·5) | 238 (26·7) | 285 (21·8) | 1112 (30·5) | 45359 (23·6) |
| People who had multiple admissions* (%) |  |  |  |  |  |  |
| No | 123140 (61·0) | 2138 (55·1) | 476 (53·4) | 767 (58·6) | 2061 (56·5) | 117698 (61·3) |
| Yes | 78723 (39·0) | 1741 (44·9) | 415 (46·6) | 542 (41·4) | 1586 (43·5) | 74439 (38·7) |
| Hospital admissions (counts) | 2314076 | 69885 | 15063 | 20396 | 60813 | 2147919 |
| Hospital admissions (mean (SD)) | 11·5 (25·9) | 18·02 (51·2) | 16·9 (45·4) | 15·6(46·7) | 16·7 (40·2) | 11·2 (24·4) |
| Multiple Hospital admissions* (counts) | 278510 | 7213 | 1641 | 2252 | 6414 | 260990 |
| Multiple Hospital admissions* (mean (SD)) | 3·54 (3·02) | 4·14 (4·23) | 3·95 (4·14) | 4·15 (4·06) | 4·04 (3·81) | 3·51 (2·94) |

Data are n (%), unless otherwise specified. *Values are in the final three months of life· QOF: Quality of Outcome Framework. IMD=Index for Multiple Deprivation

**Table S5:** characteristics of individuals who visited ED by ethnic group between 2011 to 2019

|  | Overall | Black | Mixed | Other | South Asian | White |
| --- | --- | --- | --- | --- | --- | --- |
| n | 182868 | 3719 | 845 | 1208 | 3485 | 173611 |
| Age (mean (SD)) | 77·30 (12·3) | 72·40 (14·4) | 72·04 (14·6) | 71·55 (15·4) | 71·75 (13·7) | 77·58 (12·1) |
| Age group (%) |  |  |  |  |  |  |
| 18-50 | 5492 (3·0) | 329 (8·8) | 76 (9·0) | 135 (11·2) | 286 (8·2) | 4666 (2·7) |
| 50-59 | 12854 (7·0) | 557 (15·0) | 125 (14·8) | 154 (12·7) | 430 (12·3) | 11588 (6·7) |
| 60-69 | 29907 (16·4) | 528 (14·2) | 140 (16·6) | 242 (20·0) | 739 (21·2) | 28258 (16·3) |
| 70-79 | 51540 (28·2) | 951 (25·6) | 228 (27·0) | 268 (22·2) | 959 (27·5) | 49134 (28·3) |
| 80+ | 83075 (45·4) | 1354 (36·4) | 276 (32·7) | 409 (33·9) | 1071 (30·7) | 79965 (46·1) |
| Sex (%) |  |  |  |  |  |  |
| Female | 85361 (46·7) | 1428 (38·4) | 367 (43·4) | 560 (46·4) | 1553 (44·6) | 81453 (46·9) |
| Male | 97507 (53·3) | 2291 (61·6) | 478 (56·6) | 648 (53·6) | 1932 (55·4) | 92158 (53·1) |
| Settlement (%) |  |  |  |  |  |  |
| Missing | 158 (0·1) | 3 (0·1) | 0 (0·0) | 2 (0·2) | 5 (0·1) | 148 (0·1) |
| Rural | 25389 (13·9) | 18 (0·5) | 29 (3·4) | 57 (4·7) | 33 (0·9) | 25252 (14·5) |
| Urban | 157321 (86·0) | 3698 (99·4) | 816 (96·6) | 1149 (95·1) | 3447 (98·9) | 148211 (85·4) |
| Practice Region (%) |  |  |  |  |  |  |
| East Midlands | 3315 (1·8) | 52 (1·4) | 6 (0·7) | 9 (0·7) | 53 (1·5) | 3195 (1·8) |
| East of England | 7806 (4·3) | 43 (1·2) | 23 (2·7) | 49 (4·1) | 96 (2·8) | 7595 (4·4) |
| London | 22585 (12·4) | 148 (4·0) | 42 5·0) | 74 (6·1) | 85 (2·4) | 22236 (12·8) |
| Northeast | 8110 (4·4) | 5 (0·1) | 11 (1·3) | 13 (1·1) | 19 (0·5) | 8062 (4·6) |
| Northwest | 40020 (21·9) | 198 (5·3) | 82 (9·7) | 138 (11·4) | 358 (10·3) | 39244 (22·6) |
| South Central | 36768 (20·1) | 190 (5·1) | 99 (11·7) | 198 (16·4) | 352 (10·1) | 35929 (20·7) |
| Southwest | 25500 (13·9) | 2664 (71·6) | 465 (55·0) | 598 (49·5) | 1824 (52·3) | 19949 (11·5) |
| West Midlands | 32038 (17·5) | 399 (10·7) | 111 (13·1) | 119 (9·9) | 668 (19·2) | 30741 (17·7) |
| Yorkshire & The Humber | 6726 (3·7) | 20 (0·5) | 6 (0·7) | 10 (0·8) | 30 (0·9) | 6660 (3·8) |
| Cancer primary site (%) |  |  |  |  |  |  |
| Bone | 2731 (1·5) | 44 (1·2) | 15 (1·8) | 26 (2·2) | 54 (1·5) | 2592 (1·5) |
| Multiple myeloma | 4445 (2·4) | 237 (6·4) | 44 (5·2) | 27 (2·2) | 128 (3·7) | 4009 (2·3) |
| Breast | 33708 (18·4) | 540 (14·5) | 136 (16·1) | 197 (16·3) | 649 (18·6) | 32186 (18·5) |
| Cervical | 2196 (1·2) | 53 (1·4) | 8 (0·9) | 28 (2·3) | 35 (1·0) | 2072 (1·2) |
| Colorectal | 33939 (18·6) | 525 (14·1) | 131 (15·5) | 202 (16·7) | 554 (15·9) | 32527 (18·7) |
| Head & neck | 7215 (3·9) | 89 2·4) | 26 (3·1) | 40 (3·3) | 167 (4·8) | 6893 (4·0) |
| liver | 6488 (3·5) | 135 (3·6) | 39 (4·6) | 85 (7·0) | 265 (7·6) | 5964 (3·4) |
| lung | 44815 (24·5) | 656 (17·6) | 200 (23·7) | 312 (25·8) | 824 (23·6) | 42823 (24·7) |
| Pancreas | 9162 (5·0) | 223 (6·0) | 46 (5·4) | 70 (5·8) | 205 (5·9) | 8618 (5·0) |
| prostate | 32972 (18·0) | 1023 (27·5) | 167 (19·8) | 164 (13·6) | 468 (13·4) | 31150 (17·9) |
| Stomach | 5197 (2·8) | 194 (5·2) | 33 (3·9) | 57 (4·7) | 136 (3·9) | 4777 (2·8) |
|  |  |  |  |  |  |  |
| Palliative care QoF (%) |  |  |  |  |  |  |
| No | 83145 (45·5) | 1821 (49·0) | 362 (42·8) | 530 (43·9) | 1556 (44·6) | 78876 (45·4) |
| Yes | 99723 (54·5) | 1898 (51·0) | 483 (57·2) | 678 (56·1) | 1929 (55·4) | 94735 (54·6) |
| Comorbidity Counts (%) |  |  |  |  |  |  |
| 0 | 21322 (11·7) | 526 (14·1) | 138 (16·3) | 252 (20·9) | 424 (12·2) | 19982 (11·5) |
| 1 | 36963 (20·2) | 810 (21·8) | 154 (18·2) | 316 (26·2) | 624 (17·9) | 35059 (20·2) |
| 2 | 40234 (22·0) | 802 (21·6) | 172 (20·4) | 211 (17·5) | 729 (20·9) | 38320 (22·1) |
| 3 | 34743 (19·0) | 706 (19·0) | 169 (20·0) | 162 (13·4) | 671 (19·3) | 33035 (19·0) |
| 4 | 24345 (13·3) | 421 (11·3) | 95 (11·2) | 135 (11·2) | 512 (14·7) | 23182 (13·4) |
| 5+ | 24888 (13·6) | 441 (11·9) | 116 (13·7) | 129 (10·7) | 509 (14·6) | 23693 (13·6) |
| Missing | 373 (0·2) | 13 (0·3) | 1 (0·1) | 3 (0·2) | 16 (0·5) | 340 (0·2) |
| IMD quintile (%) |  |  |  |  |  |  |
| 1 | 30318 (16·6) | 136 (3·7) | 100 (11·8) | 171 (14·2) | 320 (9·2) | 29591 (17·1) |
| 2 | 31455 (17·2) | 197 (5·3) | 82 (9·7) | 164 (13·6) | 421 (12·1) | 30591 (17·6) |
| 3 | 37803 (20·7) | 675 (18·2) | 165 (19·5) | 274 (22·7) | 660 (19·0) | 36029 (20·8) |
| 4 | 38438 (21·0) | 1282 (34·5) | 268 (31·7) | 336 (27·9) | 1019 (29·3) | 35533 (20·5) |
| 5 | 44694 (24·5) | 1426 (38·4) | 230 (27·2) | 261 (21·6) | 1058 (30·4) | 41719 (24·1) |
| People who had multiple ED visits (%) |  |  |  |  |  |  |
| No | 141210 (77·2) | 2491 (67·0) | 589 (69·7) | 895 (74·1) | 2405 (69·0) | 134830 (77·7) |
| Yes | 41658 (22·8) | 1228 (33·0) | 256 (30·3) | 313 (25·9) | 1080 (31·0) | 38781 (22·3) |
| ED Visits (counts) |  |  |  |  |  |  |
| 786143 | 22334 | 5138 | 6197 | 21141 | 731333 |  |
| ED Visits (mean (SD)) | 4·30 (4·91) | 6·01 (5·73) | 6·08 (22·6) | 5·13 (6·27) | 6·07 (6·17) | 4·21 (4·59) |
| Multiple ED Visits* (counts) | 103165 | 3227 | 686 | 822 | 2852 | 95578 |
| Multiple ED Visits* (mean (SD)) | 2·48 (0·88) | 2·63 (0·96) | 2·68 (1·56) | 2·63 (1·12) | 2·64 (1·04) | 2·46 (0·86) |

Data are n (%), unless otherwise specified. *Values are in the final three months of life· QOF: Quality of Outcome Framework. IMD=Index for Multiple Deprivation

**Figure S1:** Trends in the rate of opioid prescription by ethnic group between 2011-2020
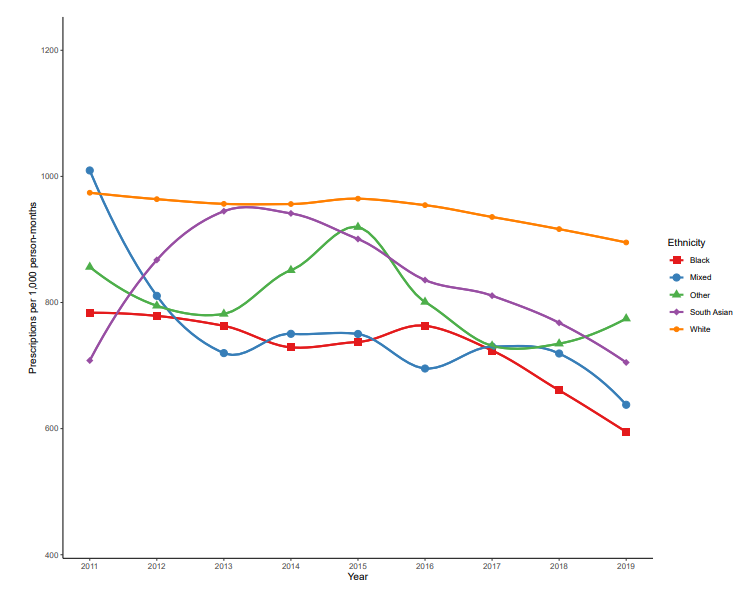


**Figure S2:** Trends in the rate of prescription of Step 2 (weak) and Step 3 opioids (strong) by ethnic group between 2011 to 2019


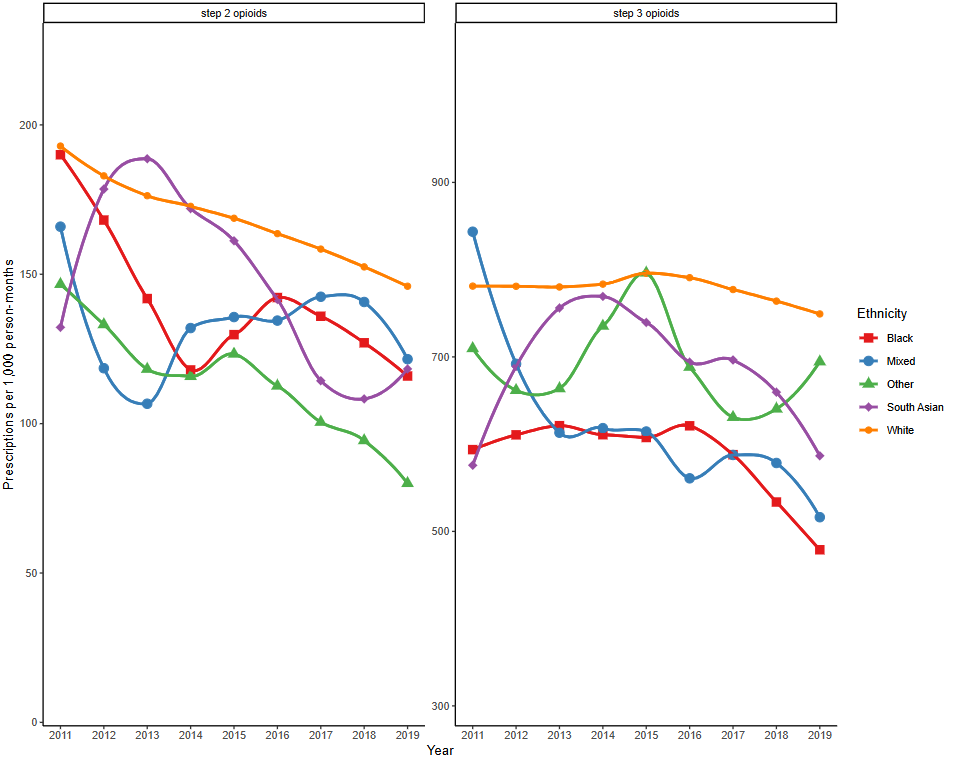


**Figure S2.** Step 3: Buprenorphine, Diamorphine, Fentanyl, Dipipanone, Hydromorphone, Methadone, Morphine, Oxycodone, Pethidine, Tapentadol, Alfentanil. Step 2: Codeine, Dihydrocodeine, Meptazinol, Pentazocine, Tramadol.

**Figure S3:** Forest plot of the association between patient ethnicity and Step 3 & 2 Opioids in the final three months of life (16 ethnic subcategories)


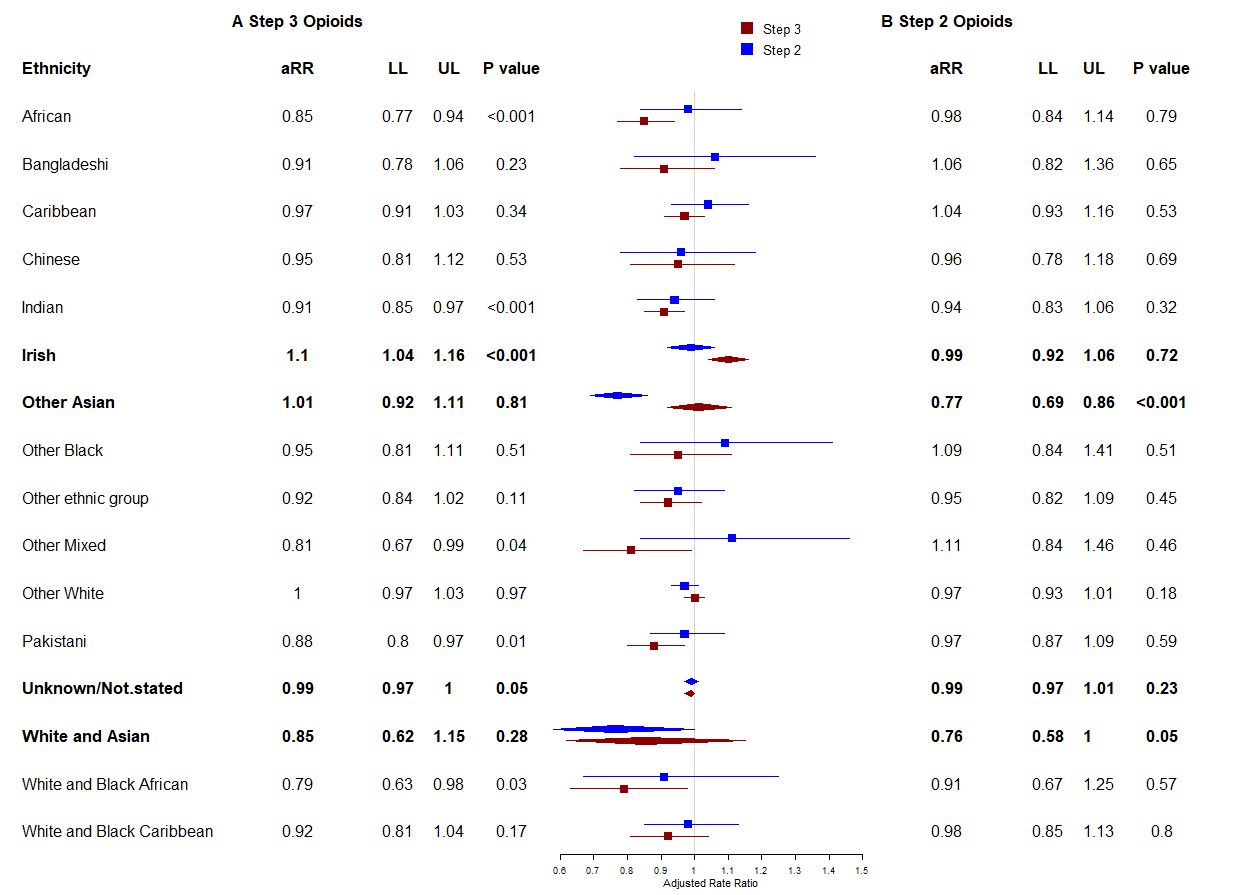


Patients from the White British ethnic group are the reference category*

**Figure S4:** Forest plot of the association between patient ethnicity and multiple ED visits and multiple hospital admissions in last 6 months of life


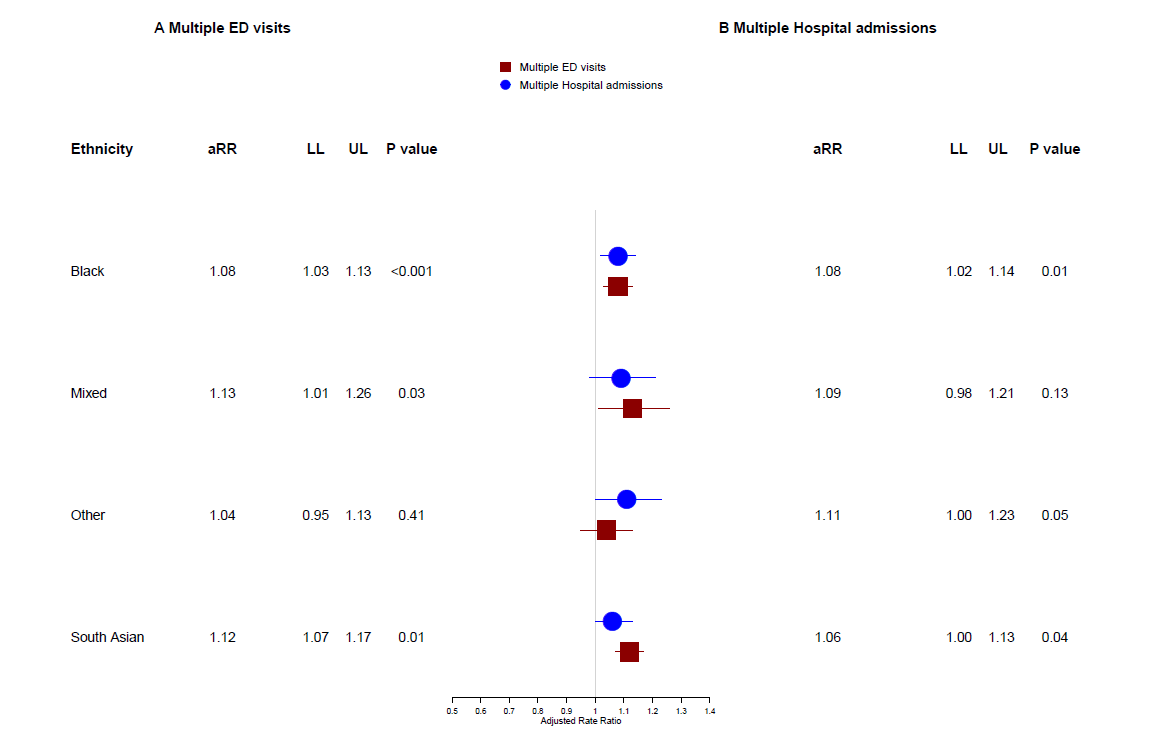


Patients from the White ethnic group are the reference category*

**Figure S5:** Forest plot of the association between patient ethnicity and multiple ED visits and multiple hospital admissions (16 ethnic subgroups) – Final three months of life


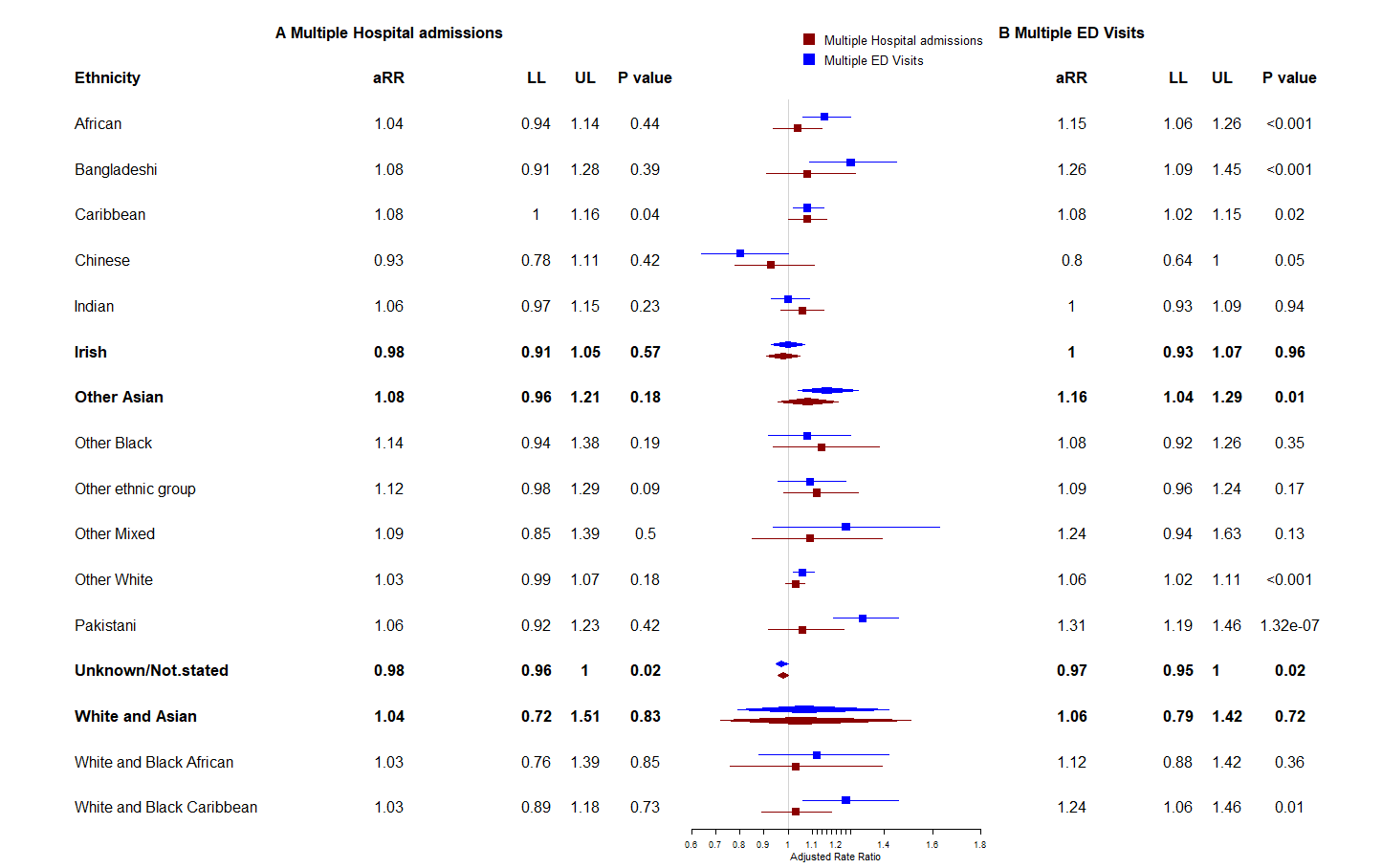


Patients from the White British ethnic group are the reference category*

Table S6 Association between prescription and patient ethnicity stratified by deprivation quintiles – final three months of life

| **Ethnicity** | **Adjusted Rate Ratio**  **(95% CI)** | **P values** | **SES quintile** |
| --- | --- | --- | --- |
| Black | 1.09(0.86-1.36) | 0.484 | 1 (Least deprived) |
| Mixed | 0.81(0.67-0.98) | 0.032 |  |
| Other | 0.79(0.67-0.94) | 0.007 |  |
| South Asian | 0.91(0.78-1.06) | 0.234 |  |
| Black | 0.82(0.67-0.99) | 0.044 | 2 |
| Mixed | 0.75(0.62-0.9) | 0.002 |  |
| Other | 1.04(0.9-1.21) | 0.606 |  |
| South Asian | 0.97(0.84-1.12) | 0.678 |  |
| Black | 0.92(0.84-1.00) | 0.05 | 3 |
| Mixed | 0.9(0.76-1.07) | 0.224 |  |
| Other | 0.82(0.74-0.92) | 0.00 |  |
| South Asian | 0.9(0.82-0.98) | 0.018 |  |
| Black | 0.91(0.84-0.99) | 0.035 | 4 |
| Mixed | 0.88(0.77-1.01) | 0.06 |  |
| Other | 1.01(0.88-1.16) | 0.908 |  |
| South Asian | 0.96(0.88-1.04) | 0.338 |  |
| Black | 0.89(0.83-0.96) | 0.002 | 5 |
| Mixed | 0.83(0.71-0.96) | 0.015 |  |
| Other | 0.86(0.77-0.97) | 0.014 |  |
| South Asian | 0.89(0.84-0.94) | 0.000 |  |

Adjusted rate ratios (aRRs) and 95% Confidence Interval (95% CI.) of the association between prescription and ethnicity stratified by deprivation quintiles (quintile 1 is the least deprived). Patients form the White ethnicity are the comparison group p. An aRR value > 1 indicates a higher rate of prescription than the reference category. Models were adjusted for patients' sociodemographic characteristics including age, cancer type, Practice-Level urban-rural classification, year of prescription issue, practice region, palliative care need and number of comorbidities.
